# Towards Clean and Safe Water: A Review on the Emerging Role of Imprinted Polymer-Based Electrochemical Sensors

**DOI:** 10.3390/s21134300

**Published:** 2021-06-23

**Authors:** Xiaofeng Zheng, Sohayb Khaoulani, Nadia Ktari, Momath Lo, Ahmed M. Khalil, Chouki Zerrouki, Najla Fourati, Mohamed M. Chehimi

**Affiliations:** 1 Université de Paris, CNRS, ITODYS (UMR 7086), 75013 Paris, France; xiao-feng.zheng@etu.u-paris.fr; 2 SATIE, UMR CNRS 8029, Cnam, 75003 Paris, France; sohayb.khaoulani@lecnam.net (S.K.); chouki.zerrouki@lecnam.net (C.Z.); fourati@cnam.fr (N.F.); 3 Laboratoire Matériaux, Traitement et Analyse, INRAP, BiotechPole Sidi-Thabet, Ariana 2032, Tunisia; nadia.ktari@inrap.mesrs.tn; 4 Département de Chimie, Laboratoire de Chimie Physique Organique & Analyse Instrumentale, Faculté des Sciences, Université Cheikh Anta Diop, Dakar 5005, Senegal; momath.lo@ucad.edu.sn; 5 Photochemistry Department, National Research Centre, Dokki, Giza 12622, Egypt; akhalil75@yahoo.com; 6 Université Paris Est, CNRS, ICMPE, UMR7182, 94320 Thiais, France

**Keywords:** imprinted polymers, electrochemical sensors, pesticides, metal ions, bacteria

## Abstract

This review critically summarizes the knowledge of imprinted polymer-based electrochemical sensors for the detection of pesticides, metal ions and waterborne pathogenic bacteria, focusing on the last five years. MIP-based electrochemical sensors exhibit low limits of detection (LOD), high selectivity, high sensitivity and low cost. We put the emphasis on the design of imprinted polymers and their composites and coatings by radical polymerization, oxidative polymerization of conjugated monomers or sol-gel chemistry. Whilst most imprinted polymers are used in conjunction with differential pulse or square wave voltammetry for sensing organics and metal ions, electrochemical impedance spectroscopy (EIS) appears as the chief technique for detecting bacteria or their corresponding proteins. Interestingly, bacteria could also be probed via their quorum sensing signaling molecules or flagella proteins. If much has been developed in the past decade with glassy carbon or gold electrodes, it is clear that carbon paste electrodes of imprinted polymers are more and more investigated due to their versatility. Shortlisted case studies were critically reviewed and discussed; clearly, a plethora of tricky strategies of designing selective electrochemical sensors are offered to “Imprinters”. We anticipate that this review will be of interest to experts and newcomers in the field who are paying time and effort combining electrochemical sensors with MIP technology.

## 1. Introduction

Human activities revolve around water, be it in industry, chemistry, agriculture or even for living. Therefore, it is quasi unavoidable that water ends up polluted [1]. Water pollution refers to all substances that human activity introduces into water, be it toxic or benign pollutants.

Water depollution is a major issue nowadays; however, in order to eliminate harmful substances, one needs to first characterize and quantify them in order to design appropriate pollutant removal methods, e.g., by adsorption, filtration or degradation. In this sense, the detection of species needs to be accurate and selective. Several methods meet these requirements, but they often require expensive materials and instruments; moreover, the measurement time is a significant criterion. Towards this end, electrochemistry is an excellent means of sensing pollutants because of its high sensitivity (it goes down to the femtomolar regime), selectivity and low cost [2]. Furthermore, the measurements are easy to set up and above all miniaturization is possible so that electrosensing could be directly done near water sources using portable devices [3]. These features are at the origin of the success story of electrochemical sensors [4,5,6,7].

Sensitivity is certainly an important characteristic of sensors and could even be a more decisive parameter. In this regard, bio-inspired molecularly imprinting polymers (MIPs) stand as excellent sensing materials. They can be prepared in bulk or as thin layers on the electrode surface, could have high sensitivity by nanostructuration and last but not least they are selective by design. Indeed, MIPs are prepared in the crosslinked form in the presence of template species (organics, metal ion, microorganism) which leave prints in the polymer network upon removal by appropriate solvents. These prints constitute the receptor sites that will recognize the template at the rebinding step, with excellent selectivity. MIP-based electrochemical sensors are increasingly employed in various domains: in medicine to detect and capture biologic molecules [8], in the food industry for quality control and food security [9,10], or to track heavy metal ions to monitor water quality [11]. Moreover, advances on transducers, for example carbon based nanostructured materials, enable achieving outstanding performances of MIPs [12]. From our perspective, with past and present active research on polymer thin film preparation, surface interactions and applications in sensor science, we wished to address the crucial point of three kinds of toxic pollutants: organics represented by pesticides (for example, the glyphosate case [13]), metal ions owing to their growing occurrence in water and soils [14], and finally bacteria due to the sanitary issues they raise [15] as well as the increasing demand for food security [16] on the one hand, and the ever-lasting problem of nosocomial diseases, on the other hand [17]. The three types of pollutants differ in composition and size, and require completely different imprinting techniques as will be discussed at length in Section 5.

## 2. Scope of the Review

We review recent advances in MIP-based electrochemical sensors of three categories of pollutants of water sources: pesticides, metal ions and pathogenic bacteria. Table 1 reports shortlisted relevant reviews, but they are either too general, or concern one kind of sensing materials or target selected series of compounds or ions, or concentrate on chemometrics for developing electronic tongues, hence the interest of the actual review paper which tackles polymerization techniques for making MIP-based electrochemical sensors relevant to environmental and life sciences.

The review is constructed with the following sections:

- Methods of synthesis of molecularly imprinted polymers
- Design of sensing electrodes
- Electrochemical characterization techniques
- Case studies of imprinted polymer-based electrochemical sensors of the target species. The focus is on imprinted vinylic, conjugated and sol-gel polymers.

There is also a growing interest in imprinted chitosan-based electrochemical sensors. The reader is referred to [22,24,25] for details.

## 3. Methods of Preparation of Imprinted Polymers and Electrode Materials

### 3.1. Monomers and Polymers

**Figure 1:**
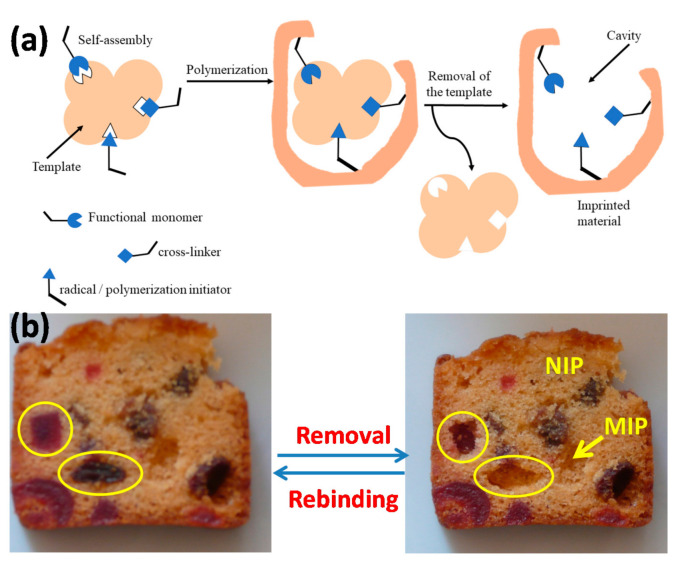
Principle of making MIPs (**a**), and illustration of the imprinting technique by digital photographs of a slice of cake before after removal of candied fruits (**b**). NIP: non-imprinted polymer.

MIPs are synthetized by (co-)polymerization of functional monomers and cross-linkers in the presence of template compounds or microorganisms (Figure 1a). Chelators could be added in the pre-polymerization mixture, in a variety of solvents [26,27]. After synthesis, templates are removed from the crosslinked polymeric matrix or coating, leaving three-dimensional cavities which are complementary in terms of shape and functional groups to the targeted compounds or microorganisms. Figure 1b illustrates the concept of molecular imprinting with a picture of a slice of cake cooked with candied fruits. Clearly, the candied fruits leave prints in the cake once removed and the shape fit in well only with the ingredient (“template”) used to cook the cake and that has been removed. Part of the cake slice that has no candied fruit corresponds to the non-imprinted polymer (NIP). At the molecular level, shape only does not suffice to have a good sensor; interfacial interactions matter very much, and this is the reason for using functional monomers that tightly bind the template molecules. MIPs were widely used as solid phase extractors and as sensitive recognition elements of chemical and biological sensors [28,29,30].

Imprinted organic and sol-gel polymers can be prepared using a large variety of monomers, the choice depending on the application (Table 2).

**Figure 2:**
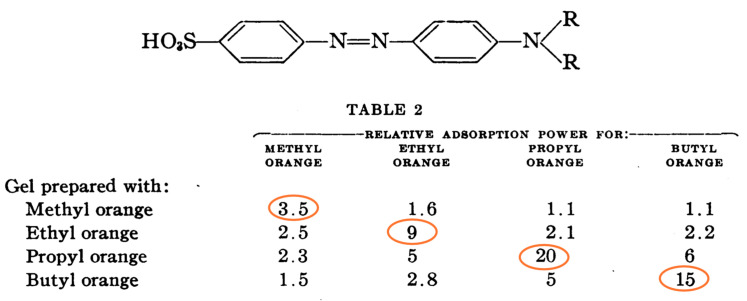
Screenshot of Dickey’s paper: Chemical structure of methyl orange and results of its relative adsorption (Adapted from [33]; paper in public domain).

Most of the studies concern the preparation of crosslinked vinylic polymers or conjugated polymers such as polypyrrole and polyaniline. It is important to note that the latter do not require crosslinking monomers as they are well-known to have rigid 3D structure, and polypyrrole is even naturally crosslinked [31,32]. Besides organic monomers, there are several reports on imprinted sol-gel inorganic polymers based on silica. As a matter of fact, we would like to recall that the very first imprinted polymers were designed by Dickey [33] (Figure 2) and concerned alkyl orange dye-imprinted silicas prepared by sol-gel chemistry. It was demonstrated that the adsorbent was selective for the uptake of the dye used in the preparation of the gel. Hence, preparation of gel using methyl orange led to selective adsorption of this dye over ethyl, propyl and butyl orange dyes. Similar trends were obtained for the three other syntheses (see circled values of relative adsorption power). The organic imprinted polymers were introduced in 1972 by Wulff and Sahran [34] to describe the concept of “enzyme-analogue built polymers”, which is decades after Dickey’s work.

#### 3.1.1. Vinylic Imprinted Polymers

Most imprinted vinylic polymer powders, nanocomposites and coatings are prepared via free radical polymerization using AIBN, Irgacure or potassium persulfate. Free radical or photoinduced radical polymerization or controlled photopolymerization (such as photo-iniferter [35,36], INItiation–TransFER–TERminaison agent) is ideal as it does not require heating and could be achieved within minutes to a few hours. In the case of grafted imprinted polymers, photopolymerization is unique for its spatiotemporal aspect. The polymer can be grafted on a selected area and growth be controlled with irradiation time. Ion imprinted clay-polymer nanocomposites have recently been prepared by radical photopolymerization under visible [37] or UV light [38] using Type II photoinitiators. For grafted MIP thin films prepared by surface confined photopolymerization, the reader is referred to [39,40].

**Figure 3:**
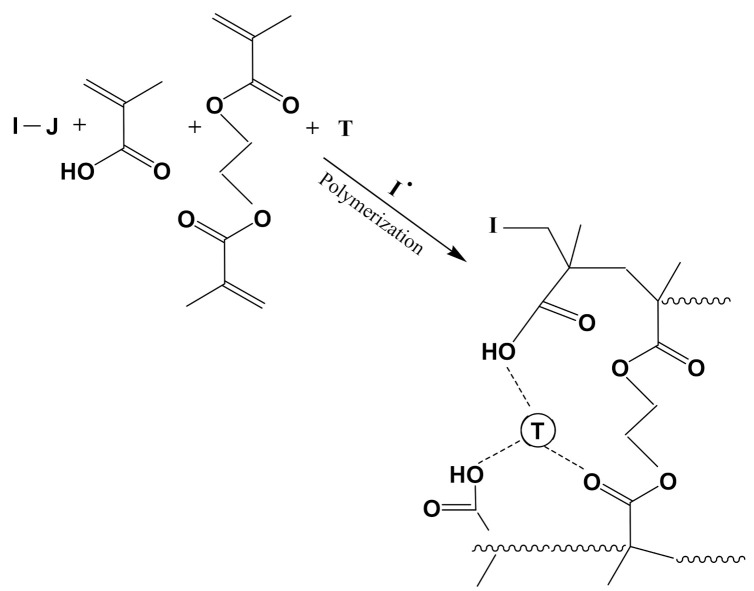
Simplified mechanism of imprinted vinylic polymer synthesis by radical polymerization. Example is given for methacrylic acid functional monomer and ethylene glycol dimethacrylate (EGDMA) crosslinker. I–J is the initiator and T the template.

Figure 3 shows a simplified mechanism of radical polymerization whether it is of the free radical or controlled type. Initiator is activated thermally or photochemically and the initiating radical triggers polymerization of the prepolymerization complex (PCC) to yield, after a few hours or even better a few minutes (in the case of photopolymerization), 3D crosslinked polymers with entrapped template species (T). Precipitation polymerization requires crashing of the former monolith, but, in the case of the synthesis of imprinted thin polymer film, it is essential to first prepare graft initiators to the electrode in order to confine polymerization to the surface and limit precipitation of imprinted polymers or interpenetration of grafted polymers with free crosslinked polymers; this makes cleaning the grafted imprinted polymer tedious. For example, Type II radical photoinitiators are preferred over Type I phtoinitiators as they drastically limit bulk solution polymerization. Type I photoinitiators give two radicals upon thermal or photo-clevage: initiating radicals in solution and at the surface.

As far as electrochemical sensors are concerned, numerous studies covered the nanostructuration of vinylic imprinted polymers by metallic or carbon nanoparticles [41,42], which can be coated on free electrode surfaces or mixed with graphite in order to prepare carbon paste electrodes. Such nanostructuration enhances the conductivity of the polymer and facilitates electron transfer.

#### 3.1.2. Conductive Polymers

Conducting polymers (CPs) are π-conjugated organic materials with electrical and optical properties comparable to those of inorganic semiconductors and metals. They can be synthesized using cost-effective, simple and versatile approaches. Several methodologies have been developed to prepare CPs (precipitation polymerization, electropolymerization, sonochemical synthesis and photopolymerization). Electropolymerization remains, however, the most investigated technique as it permits to address the morphology, thickness and conductivity of CPs, and is suitable for electrochemical sensors [43]. These features led to increased applications of CPs in the fabrication of chip-based sensors, biosensors, diagnostic and environmental monitoring devices [44,45,46,47]. However, with the development of conductive polymer nanocomposites, oxidative chemical polymerization of conjugated monomers is versatile because the same nanocomposites could be employed as adsorbents or mixed with graphite powder to make carbon paste electrodes for selective electrochemical sensors. If the chemical method could require one or two hours, electropolymerization is most probably the fastest process among all others discussed in this review. Moreover, electropolymerization can conducted in aqueous media, in a supporting electrolyte with or without ligand, if IIP is meant to be prepared.

**Figure 4:**
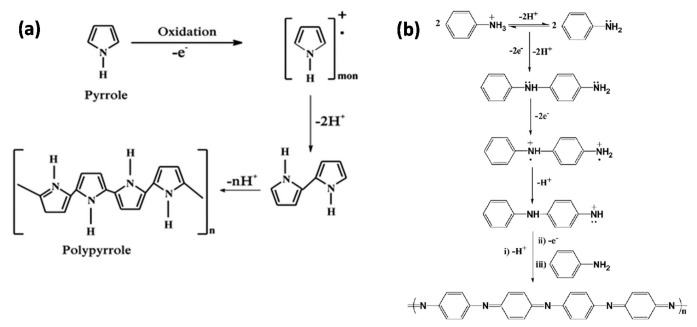
Simple pathways for the synthesis of polypyrrole (**a**) and polyaniline (**b**). Figure 4b is reproduced from [48] with the permission of Elsevier.

Figure 4 depicts general pathways for polypyrrole and polyaniline syntheses. Bearing N-H groups, these polymers have the ability to interact with the template in the course of the polymerization process.

#### 3.1.3. Sol-Gel Synthesis

**Figure 5:**
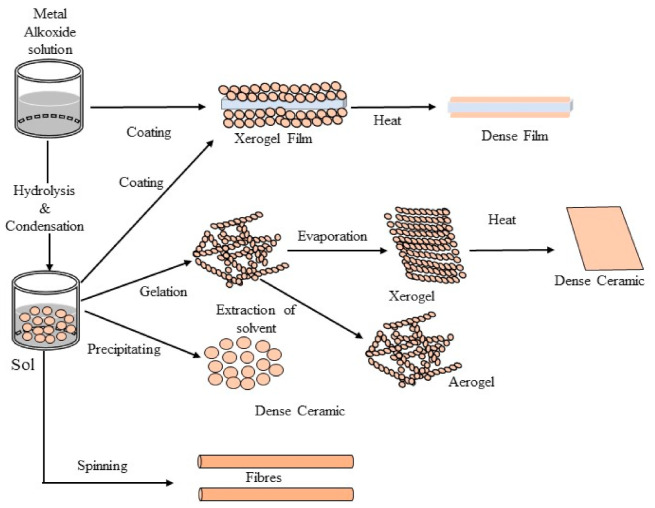
Sol-gel methods for nanoparticle synthesis.

The sol-gel process has been used intensively for the synthesis of porous nanostructures mainly composed of transition metal alkoxides and siloxane (Si−O) backbone structure [49]. Three reaction steps are generally involved during a given sol-gel procedure: (i) interaction between metal cations and water molecules, (ii) hydrolysis of silicone monomers, and (iii) polycondensation of the silica into a porous 3D network. Sol-gel chemistry provides a relatively simple way for the design of electrochemical sensing layers [50,51,52]. A schematic representation of sol-gel methods is depicted in Figure 5.

### 3.2. Electrode Material Preparation

**Figure 6:**
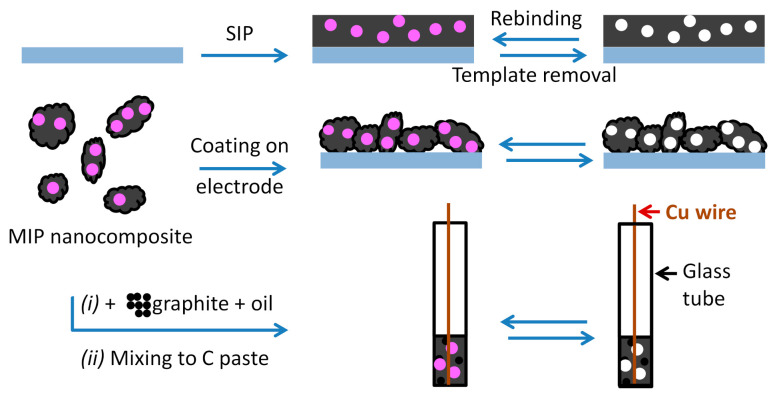
Three main methods to prepare MIP-based electrodes for electrochemical sensors, by direct surface initiated polymerization (SIP) on the electrode chemical (**top**), by preparation of MIP nanocomposite and coating it on the electrode surface (**middle**), and by preparing carbon paste electrode (CPE) using a mixture of MIP and carbon powders in mineral oil. MIP designates pure imprinted polymer or its corresponding composite containing nanostructures (clay, carbon, nanometal…). For the sake of simplicity, MIP means either a molecular, ion or pathogen-imprinted polymer.

For the purpose of electrochemical sensors, there are three main options for making MIP sensing layers and composites (Figure 6): (i) polymerization conducted directly on the transducer surface, (ii) preparation of MIP or imprinted nanocomposite as powder that is coated on the electrode surface, and (iii) preparation of carbon paste electrode (CPE) from the mixture of carbon and MIP powder. “Imprinters” are interested more and more in CPEs due to their flexibility, low cost and good electrical conductivity [53].

Whilst preformed or in situ synthesized polymers can be deposited on the bare transducer, surface-initiated polymerization, in particular, requires the use of a coupling agent in order to covalently link the MIP to the transducer. In this regard, silane, thiols and aryl diazonium coupling agents were successfully employed. Indeed, they are bi-functional compounds and bear reactive groups to bind preformed MIPs, or even a polymerization initiator group to trigger surface polymerization. Silanes are mostly applied to metallic surfaces with a thin oxide film or to nanoparticles, whereas thiols are frequently used to modify gold electrodes. In the recent years, diazonium salts appeared as the most versatile coupling agents due to their ability to bind many more surfaces such as metals, oxides, sp^2^ and sp^3^ carbon allotropes, insulating polymers and transparent semi-conductor electrodes, to name only some of these materials. Furthermore, diazonium salts could be easily produced from aniline derivative precursors, bearing numerous functional groups for the covalent attachment of polymers. For these reasons, the surface and interface chemistry of diazonium salts is particularly suitable for making MIP-based electrochemical sensing layers with a robust electrode-MIP interface [39,54,55].

There are four polymerization methods that are compatible with the design of MIP-based sensors: thermal or controlled radical polymerization (CRP), radical photopolymerization, (electro)polymerization of conjugated monomers and sol-gel polymerization.

Atom Transfer Radical Polymerization (ATRP) is the most applied type of CRP [54] (The combination of “molecularly imprinted polymers” and “atom transfer radical polymerization”, “reversible addition-fragmentation chain transfer”, “inferter” (initiator-transfer-terminator), and “Nitroxide-mediated radical” polymerization returned 144, 119, 72 and four publications, respectively. Web of Science, last accessed 9 June 2021), with less specific restrictions nowadays, as it could be conducted in air [56] and tolerant to most important vinylic monomers employed in MIP technology. In order to polymerize directly on the transducer, the initiator of polymerization needs to be grafted onto the metal surface [57].

Electropolymerization is related to conjugated monomers; initiation of the polymerization is a monomer oxidation process which leads to the formation of a radical cation. Then, two oxidized monomers form a dimer; the polymerization goes on with the addition of other monomers. The final polymer coating is electrically conductive. The rationale for making MIPs by electropolymerization lies in the fact that the technique permits to synthesize the thin film, to characterize its redox properties and to use the same electrode and the same apparatus for electrosensing measurements. Electropolymerization is also suitable for nanostructuration of MIP films [14,58]. This is particularly important for directly coating electrodes at the polymer synthesis stage.

Sol-gel polymerization consists of creating a solution of monomers and silica; gelling of the solution on a surface will yield the polymer film. This method is extremely powerful in creating films and coatings [59].

Photopolymerization requires UV or visible light in order to trigger radical polymerization. Type I photoinitiators (e.g., Irgacure) are not very suitable because most of the polymers are synthesized in bulk even though the initiator is attached to the surface [60]. Instead, a Type II initiating system consists of an attached radical polymerization photoinitiator (e.g., *N*,*N*-dimethylaminophenyl group or a derivative) to the surface and a co-initiator that acts as hydrogen abstractor, for example benzoquinone (active under UV light) [61] or camphorquinone (active under visible, blue light) strips a hydrogen from the attached molecules, therefore yielding a surface bound radical that triggers the polymerization process [37].

**Figure 7:**
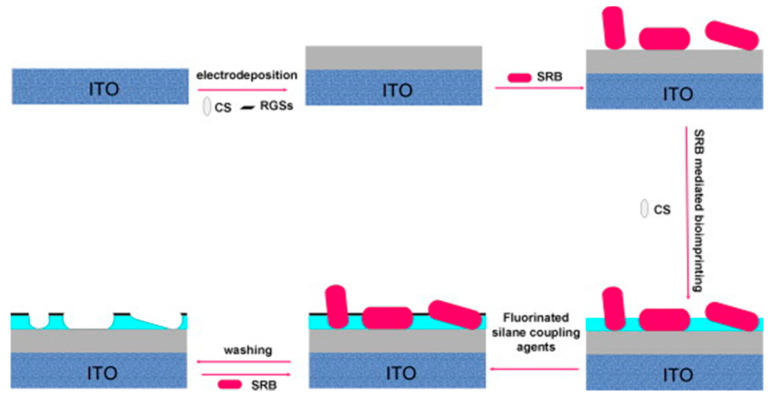
Methods of bacteria imprinting: surface imprinting of bacteria. Reproduced with permission of Elsevier from [63].

MIPs are compatible with species of molecular (e.g., bacterial signaling molecule) or macromolecular size (e.g., proteins); however, pathogens are much larger; the removal of the template after polymerization is almost impossible with macromolecules or cells without damaging the polymer network. To adapt MIP methods to pathogen imprinting, the so-called surface imprinting technique is recommended. In this strategy, only a part of the surface of pathogen is imprinted onto the polymer free surface [62]. The strategy is illustrated in Figure 7 and more detailed in Section 5.3.

## 4. Detection Methods

Electrochemical sensing techniques are among the most sensitive, simple, robust, and accurate transduction techniques. Efforts have been recently done to enable in situ multiple measurements, which has opened avenues to several chemical and biological applications, mainly the detection of harmful substances in water. Electrochemical sensors can be designed for all types of ions and molecules independently of their electroactivity. Actually, if the desired target is not electroactive, the MIP-based sensor strategy will be based on the attenuation of the electrochemical signal of a probe present in the solution [64].

**Figure 8:**
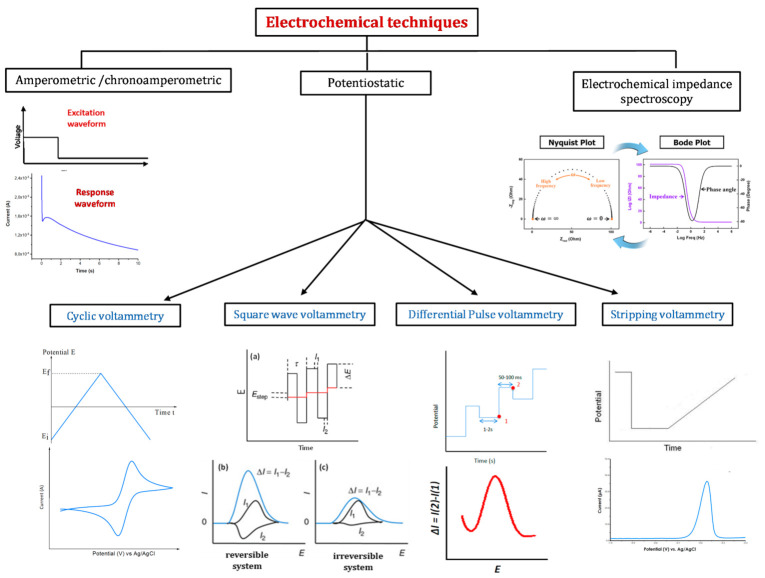
Schematic representation of the most common electrochemical techniques used in the detection of pollutants in water sources. (**a**) Details of the application of pulses in the square wave voltammetry technique and the corresponding voltammograms for reversible (**b**) and irreversible systems (**c**).

A schematic representation of the physical principles and corresponding voltammograms of the most common electrochemical techniques, used for pollutants’ detection in water sources, is depicted in Figure 8. Details are presented in the following paragraphs.

### 4.1. Cyclic Voltammetry

Cyclic voltammetry (CV) is a popular electrochemical technique usually used for quantitative analysis and for the investigation of the reduction and oxidation processes of electroactive species. During the experiment, the capacitive and faradaic currents are combined to get the total measured current. Scan rate is one of the key parameters of this technique, as it controls the “speed” of the applied potential. High scan rate values lead in fact to a decrease of the diffusion layer’s size and thus to the current’s increase. Since the high capacitive current interferes with the sensitive faradaic current (proportional to the analyte concentration in the linear domain), the technique’s sensitivity is limited [65]. CV is thus usually used to check the state of the sensors’ surfaces before and after the functionalization steps.

For more quantitative measurements, potential pulse methods, such as pulse differential voltammetry and square wave voltammetry, are generally used. These methods allow for reducing the contribution of the capacitive current, and thus to increase the sensitivity of the designed sensors.

### 4.2. Differential Pulse Voltammetry

Differential pulse voltammetry (DPV) is based on the application of a first potential value in a region where no faradaic reaction can occur. A linear slope potential is then applied to the electrode with constant amplitude potential pulses. The recorded current is calculated from the difference between the currents measured immediately before and after the application of the pulse (Figure 8). A current peak is thus displayed for a given electrochemical reaction. The DPV technique is more sensitive than the linear sweep methods since the capacitive current is minimized.

### 4.3. Square Wave Voltammetry

Square-wave voltammetry (SWV) is one of the fastest and most sensitive electrochemical techniques. The obtained limit of detection (LOD) can usually be compared to those obtained by spectroscopic and chromatographic methods [65]. Details of the application of pulses in the SWV technique, for both reversible and irreversible systems, are presented in Figure 8 (the scheme relative to SWV is adapted from [66]). The Faradaic current Δ*i* is calculated as the difference between currents measured at the end of each potential step. As the frequency is equal to 1/τ and the timing for the SWV experiment is equal to τ/2, a frequency increase leads to a τ decrease, and thus to the faradaic current increase. The resulting voltammogram peaks are then sharper and better defined [67]. SWV also permits monitoring the kinetics of reaction of a considered system. The signal-to-noise ratio increases as the square root of the scan rate. Faster analysis speed results in less consumption of electroactive species and fewer electrode surface blockage problems.

The obtained current-potential curves are generally symmetrical and have well-defined pick profiles corresponding to the oxidation and/or reduction of the electroactive species at the surface of the electrode. When the two electrochemical processes are present in the same experiment (reversible system), the curve displays the difference between the two measured currents. Hence, for a reversible system, the current obtained is much higher.

### 4.4. Amperometric Methods

In the amperometric technique, a steady potential is applied to the electrode system, and a resulting current is recorded (Figure 8). This current corresponds to the electrochemical reactions which take place on the surface of the working electrode. It can thus be used to control or quantify the involved reactions.

One of the main advantages of amperometry is providing a current measurement that varies linearly with the concentration of the analyte of interest. The response is often fast with good reproducibility and high sensitivity. Furthermore, amperometric sensors may be miniaturized and used for online monitoring. Consequently, amperometry techniques were used to develop the majority of existing portable sensors.

### 4.5. Stripping Voltammetry

Stripping (or pre-concentration) techniques have been used to detect cations, some anions and neutral species. The electrochemical procedure is carried out in two or three steps: (i) adsorption of species or their deposition occurs, during a defined time, on the electrode (pre-concentration step). The applied potential is usually controlled or fixed at the open circuit potential; (ii) the sensing electrode with preconcentrated species could be transferred to another analyte-free electrolyte or kept in the same preconcentration medium for next step; and (iii) oxidation or reduction of the accumulated species at the electrode by varying the applied potential and recording a current peak that is proportional to the concentration of these species.

The most commonly used stripping techniques are: (i) anodic stripping voltammetry (ASV), generally used for trace detection of metals such lead, copper, zinc, and cadmium; (ii) adsorption stripping voltammetry (AdSV), commonly used to detect trace amounts of cobalt, nickel, and some organic compounds; and (iii) cathodic stripping voltammetry (CSV) investigated for the detection of ionic species like selenium, sulfide and thiocyanate.

The use of pulse techniques can substantially lower LODs of the ASV technique and increases the sensitivity. The two most commonly used pulse techniques are differential pulse and square wave anodic stripping voltammetry (DPASV and SWASV, respectively). A previous work describes in more detail these different techniques for the analysis of heavy metals [68].

### 4.6. Electrochemical Impedance Spectroscopy

Electrochemical impedance spectroscopy (EIS) is a technique used to study a large variety of interfacial phenomena such as corrosion surface reactions and studies of electrochemical processes occurring in the interface between the electrode and the electrolyte solution [69,70]. It is based on the application of a small sinusoidal voltage to the working electrode and measuring the complex impedance at the electrode/electrolyte interface over an appropriate frequency range: Z(ω) (ω = 2πf is) [71]. Other configurations, with four or two electrodes, can be used depending on the envisaged application. Set-up with four-electrodes allows for follow-up electric conductivity changes in a given medium [72,73], while that with two-electrodes is often used to design capacitive affinity sensors [70] or to detect electric conductivity changes due to material and fluid properties. The impedance spectrum can be represented in two different ways (Figure 8): The “Nyquist plot”, which uses Cartesian coordinates to represent the real and imaginary parts of Z(ω), and the “Bode plot”, where both phase and log of the total impedance log Z are plotted as a function of the log of the frequency. The Bode model is particularly useful for monitoring phase regions of disturbances that are dominated by resistive or capacitive. The most popular Nyquist plot generates typical configurations according to a predominant electrochemical mechanism in an equivalent circuit model [74]. The diameter of the semicircle can represent either charge transfer, mass-transfer or pore resistances [75,76].

EIS-based sensors have been reported for numerous applications such as the detection of toxins, polluting agents, water contamination, bacteria, cancer, and other disease biomarkers [77,78]. Moreover, several biosensors with non-electroactive biological recognition elements have been designed using EIS as the transducing method. EIS provides a great advantage for affinity-based biosensors by facilitating the use of direct label-free electrochemical detection making the analysis easier, faster, and low cost.

### 4.7. Comparison of Electrochemical Techniques

Comparison between the principles features and applications of the presented electrochemical techniques is presented in Table 3.

In addition to these dynamic electrochemical techniques, the potentiometric analytical methods, based on the use of potentiometric ion-selective electrodes (ISEs) and pH electrodes, are widely investigated for pollutants tracking in water stream. These miniaturized and cost-effective electrodes offer several advantages, such as in situ measurements and more importantly selectivity for ISEs.

Historically, measurements with ISEs were done at an open circuit potential, but over the last few decades, the application was extended by applying an external current or potential control [79]. These tools are investigated in the detection of a large variety of contaminants such as ammonium [80], copper sulfate [81] and heavy metals [82].

Acidity of water is also of prime importance in both environmental and industrial fields. In fact, pollutants and contaminants can cause corrosion and thus vast damage in physical facilities like power generation water cycle chemistries or pipeline systems [83]. Electrochemical pH sensors are thus widely used to monitor pH in simple and complex real media [84].

## 5. Applications of Imprinted Polymer-Based Electrochemical Sensors

### 5.1. Tracking Pesticides with Molecularly Imprinted Polymers

#### 5.1.1. Pesticide Imprinted Sol-Gels (PISGs)

Various sol-gel based sensors were realized for pesticide detection and quantification. Beduk et al. have designed an inkjet-printed ZnO sol-gel modified PEDOT:PSS/Nafion disposable sensor for the selective detection of hydrazine [85]. Chronoamperometry and cyclic voltammetry techniques were investigated for the determination of low concentrations of hydrazine and for selectivity tests. Results indicate that the oxidation of hydrazine is catalyzed by ZnO particles, and that the modification of a PEDOT:PSS surface with ZnO sol-gel improves the sensor sensitivity and stability. LOD and sensitivity values of the designed sensor were of the order of 5 μM and 0.14 μA·μM^−1^·cm^−2^, respectively.

Organophosphorus (OPs) compounds were extensively used as insecticides, fungicides and herbicides. Exposure to OPs inhibits the activity of acetylcholinesterase (AChE), an enzyme which plays a key role in the appropriate functioning of the central nervous system [86]. OPs pesticides act generally as anti-AChE causing over-accumulation of acetylcholine and thus cholinergic toxicity [87]. Several electrochemical biosensors, based on inhibition of AChE, were thus designed for OP detection [88].

Hu et al. designed an AChE sensor based on a titanium dioxide (TiO_2_) sol-gel carrier for dichlorvos (2,2-dichlorovinyl dimethyl phosphate, DDVP) detection by CV and DPV [89]. In this study, a solution of TiO_2_ and chitosan (CS) was drop-coated on the surface of a glassy carbon electrode. After drying in air and further formation of a thin film on the surface of the electrode, a solution containing AChE and CS (0.5%) is dropped on the functionalized CGE. The designed biosensor exhibits a linear response in the concentration interval 1.13 nM to 22.6 μM, and an LOD of the order of 0.23 nM.

Cui et al. [90] designed an electrochemical AChE biosensor for the detection of an OP model, dichlorvos (DDVP) in cabbage juice samples. In this study, an rGO/GC electrode was coated with TiO_2_-CS solution and then left in air for gelation. This step was followed by the electrodeposition of a chitosan layer prior to the immobilization of AChE (in a PBS solution containing 1% bovine serum albumin). DPV results indicate that the linear range was from 0.036 μM (7.9 ppb) to 22.6 μM and that its LOD was equal to 29 nM (6.4 ppb).

DDVP detection was also investigated by Zhang et al. [91] who have designed an electrochemical biosensor based on silver nanowires (AgNWs)/glassy carbon, TiO_2_ sol-gel–CS, graphene and AChE. The electrochemical activity of this biosensor was found to be dependent on the oxidation of thiocholine (TCl), an enzymatic product obtained from AChE hydrolysis. Subsequently, TCl oxidation was investigated by DPV. This biosensor was found to be stable and selective in the presence of several interfering species; LOD was ~7.4 nM (1.64 ppb).

Song et al. [92] employed a strategy based on citrate-capped gold nanoparticles (AuNPs)/(3-mercaptopropyl)-trimethoxysilane (MPS)/Au electrode for the selective detection of carbamate. The authors reported a 3D-MPS sol–gel network which was assembled on the Au electrode surface via Au-S bond. The CV response of the biosensor was found dependent on the activity inhibition of AChE in the presence of the carbaryl(1-naphthyl methylcarbamate). This sensor revealed a linear range from 0.003 to 2 mM, an LOD of 1 nM and a sensitivity of 32.0 μA·cm^−2^·mM^−1^. The proposed biosensor shows good reproducibility and long-term storage stability.

Maulidiyah et al. [93] modified a carbon paste electrode (CPE) with a TiO_2_ sol-gel for fipronil detection in real samples. To prepare the working electrode, the authors mix TiO_2_ nanoparticles (obtained from the crush of anatase crystal) with carbon and paraffin oil, prior to heating at 80 °C. After that, the composite was entered in probe glass connected by Cu wire as a conductor and also tip electrode. Cyclic voltammetry results indicate that the TiO_2_-CPE sensor presents an LOD of 34.0 × 10^−5^ µM and 23 days of lifetime.

**Figure 9:**
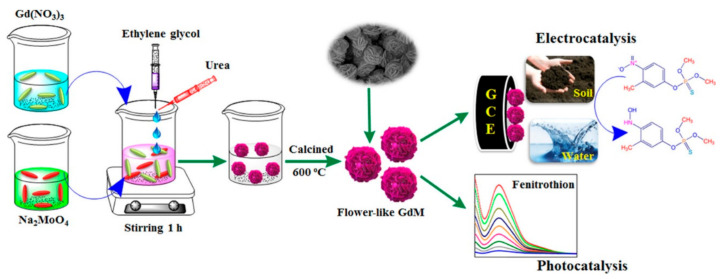
Overall synthesis procedure of flower-likeGDM and its electrocatalytic and photocatalytic applications. Reproduced with permission of ACS from [94].

Vinoth Kumar et al. [94] prepared, via a simple sol−gel technique, a 3D flower-like gadolinium molybdate (Gd_2_MoO_6_; GdM) and used it as a bifunctional catalyst for photocatalytic degradation and electrochemical detection of fenitrothion (FNT). The synthesis procedure and the further applications are presented in Figure 9.

DPV measurements indicate that the GdM catalyst plays a significant role in the electrochemical reduction of FNT. The flower-like GdM-modified GCE exhibits a wide linear range (0.02−123; 173–1823 μM), a sensitivity of the order of 1.36 μA·μM^−1^ cm^−2^ and an LOD of 5 nM. Additionally, the GdM photocatalyst could degrade above 99% of FNT under UV light irradiation with good stability even after five cycles.

We et al. [95] modified glassy carbon electrodes (GCEs) with multi-walled carbon nanotubes (MWCNTs)@TiO_2_ and Carboxymethyl chitosan (CMCS) to detect trichlorfon pesticide in fruits. The optimum mass ratio composition was found equal to 10/10/80 for MWCNTs/TiO_2_/CMCS. The analytic performances of the designed sensor were investigated by cyclic voltammetry and differential pulse voltammetry. Electrochemical results indicated a wide linear range, from 10^−5^ to 10^−11^ mol·L^−1^, a sensitivity of 0.5077 µA·M^−1^, an LOD on the order of 4 × 10^−7^ mol·L^−1^ and a recovery of 98%.

#### 5.1.2. Pesticide Imprinted Vinylic Polymers (PIVPs)

The literature reported the design of MIP based electrochemical sensors for selective detection of cypermethrin (CPM), a synthetic pyrethroid pesticide widely used in agriculture and spot treatment for insects’ control. CPM may, however, induce neurotoxicity by modulating the level of gamma-amino butyric acid [96]. Leepheng et al. [97] designed a molecularly imprinted electrochemical sensor for CPM detection in vegetable juice. In this work, the authors used methyl methacrylate (MMA), ethylene glycol dimethacrylate (EDGMA), AIBN, as the functional monomer, the cross-linker and the initiator, respectively. The polymerization was carried-out at 70 °C for 2 h. This step was followed by dropping CPM-MIP onto screen-printed electrodes (SPE) at a controlled temperature of 60 °C for 90 min. The templates were removed by ethanol and deionized water. Cyclic voltammetry measurements indicate that the CPM-MIP/SPE sensor presents an LOD of 15 ppb and a sensitivity of 0.094 μA·ppm^−1^.

Cypermethrin (CYP in this study) detection was also reported by Li et al. [98] who prepared a solution of Ag-N@ZnO/CHAC from coconut husk (CHAC), dropped it on the surface of a glassy carbon electrode and dried it under infrared lamp, prior to the MIP electro-polymerization. Two functional monomers were investigated in this study: resorcinol and dopamine. CYP extraction was performed by the immersion of the coated electrode in 0.1 M NaOH and by scanning between −1.0 V and +1.0 V for 10 cycles. CV measurements were performed to investigate the effects of double monomers use, to optimize the ratio between template and monomers, to determine the analytical performances of the sensor and for selectivity tests. The designed MIP based sensor was selective of CYP and presents an LOD on the order of 6.7 × 10^−14^ M.

Glyphosate (Gly), a synthetic herbicide, is probably the most widely used pesticide worldwide. In 2015, the World Health Organization’s International Agency for Research on Cancer classified glyphosate as potentially carcinogenic to humans. This health concern has motivated the realization of numerous chemical sensors to track glyphosate in water samples, soil, air and body fluids. In the field of MIP-based electrochemical sensors, Zouaoui et al. [99] designed a sensitive and selective sensor, in which a chitosan (CS)-Gly-MIP was electrodeposited, by cyclic voltammetry (from −1.5 to 0.5 V at a scan rate of 80 mV/s) onto a gold microelectrode surface. Cross-linking of the polymeric matrix was performed by incubating the (GLY +CS)/Au in a solution of H_2_SO_4_ for 1 h. Gly template extraction was done by incubating the microelectrodes in a protic solution acetic acid/methanol (1:1, *v*/*v*) for 30 min. The sensing properties of the designed sensor were followed-up by electrochemical impedance spectroscopy and cyclic voltammetry. Results indicate an LOD of 0.001 pg/mL and a linear range from 0.31 pg/mL to 50 ng/mL.

Glyphosate detection was also investigated by Mazouz et al. [13] who have designed an electrochemical sensor functionalized with polypyrrole (PPy)-MIP electrodeposited by chronoamperometry (CA) on the surface of gold electrodes. In order to reduce the oxidation potential of pyrrole during MIP elaboration, a thin polypyrrole blocking layer was deposited on Au surfaces. Here, Gly templates extraction was also done by incubating the electrodes in a protic solution acetic acid/methanol (1:1, *v*/*v*) for 30 min. SWV was investigated to optimize the MIP synthesis and to determine the metrological performances of the designed sensor. The sensitivity was equal to (75 ± 41) µM/nM, and the LOD was on the order of 1 pM. The dissociation constants, related to the affinity between PPy and glyphosate, were calculated from the fit of the calibration curve with a combined one site/Hill model. They were found to be Kd_1_ = (0.7 ± 0.3) pM and Kd_2_ = (1.6 ± 1.4) µM, which indicates a high affinity between Gly analytes and the cavities created in the PPy polymeric matrix.

MIP-based electrochemical sensors were also designed for the detection of organophosphorus compounds. Aghoutane et al. [100] designed an acrylamide-MIP on screen-printed gold electrodes to quantify malathion (MAL) in olive fruits and oils. In this study, a solution of MAL templates and bisacrylamide monomers was incubated at 4 °C during 6 h in the presence of N, N, N, N-tetramethyl ethylenediamine and ammonium persulfate as catalysts. Polymerization was carried out overnight in an oven at 74 °C. The template was extracted in methanol/acetic acid mixture (9/1: *v*/*v*) for 10 min. Cyclic voltammetry, DPV and EIS were used to investigate the analytical performances of the sensor. The MIP-based device exhibited satisfactory selectivity, a dynamic concentration range of (0.1–1000 pg·mL^−1^), an LOD of 0.06 pg·mL^−1^ and a recovery rate of 87.9%.

Hassan et al. [101] reported methyl parathion electrochemical detection in fish by pre-concentrating the pesticide on magnetic MIP and further readout on magneto-actuated electrode by square wave voltammetry. Magnetic-MIP was prepared using Fe_2_O_3_ as magnetite nanoparticles core, methacrylic acid as a functional monomer, EGDMA as crosslinking monomer, AIBN as a radical initiator and methyl parathion as a template. Methyl parathion extraction was done using a Soxhlet and methanol/acetic acid (9/1). SWV results indicate that magnetic-MIP/m-GEC sensor presents an LOD of 1.22 × 10^−6^ mg L^−1^ and recovery values ranging from 89.4% to 94.7%.

Wang et al. [102] fabricated a molecularly imprinted electrochemical sensor for methyl parathion (MP) quantification in vegetables and fruit matrixes. The sensor was designed using GCE and AuNPs to improve the electrical conductivity and enhance the electron transfer. The imprinted sensor was prepared in acetate buffer solution containing quercetin, resorcinol, KclO_4_, and methyl parathion templates. The composite was after that electroplated, onto the Au/GCE electrode surface, by cycling in the potential range from −0.2 to 0.9 V at a scan rate of 0.05 V/s. MP template extraction was done by submerging the electrode in ethanol acid solution for 5 s. CV Electrochemical results indicated an LOD of 0.01 μM, a good selectivity and a recovery range from 87.7 to 124.8%.

MP detection was also investigated by He et al. [103] who have synthesized the MIP by free radical polymerization in chloroform. Zinc porphyrin, EGDMA and AIBN were used as functional monomer, cross-linker and initiator, respectively. Under the optimized experimental conditions, DPV results indicate that the sensor presents an LOD of 31.6 nM and that it is stable over 30 days.

Xu et al. [104] realized a disposable electrochemical sensor for sensitive and selective detection of phosalone insecticide in agricultural products and environmental samples. A home-made carbon paste microelectrode (CPME) was modified with Zr−based metal−organic framework catalyst (Pt−UiO−66) and a mesoporous MIP. The latter was synthesized onto Pt-UiO-66/CPME by electropolymerization and a subsequent sol−gel process. SWV results revealed that a mixture of acetonitrile/methanol (1:1, *v*/*v*) can efficiently extract the templates from the polymeric matrix and that the designed sensor exhibits a linear range in the domain 0.50 nM–20 mΜ and an LOD of 0.078 nM.

**Figure 10:**
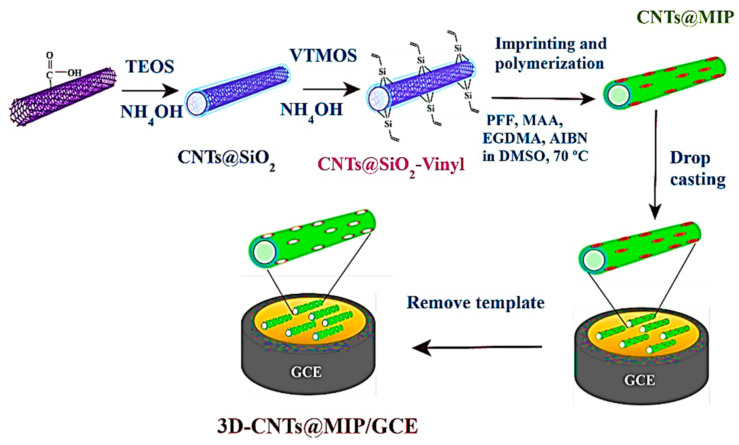
Schematic representation of the 3D-CNTs@-MIP preparation and further fabrication of the MIP sensor. Reproduced with permission of Elsevier from [105].

Amatatongchai et al. [105] designed a selective profenofos sensor in which a GCE was modified with SiO_2_-vinylcarboxylated carbon nanotubes (CNTs) and then with molecularly imprinted polymer shells. The synthesis procedure is presented in Figure 10.

The 3D-CNTs@MIP sensor exhibited a wide linearity range (01–200 μM), a low LOD, 2 nM, and a linear sensitivity, calculated from the slope of the amperometric response of 0.573 A·M^−1^.

The Zhang et al. [106] study is related to the detection of imidacloprid residue with an MIP for which the functional monomer was p-vinylbenzoic acid (VBA), and the crosslinker was EGDMA. The designed sensor was fabricated using graphene and modified glassy carbon electrode to improve the stability and the imprinting of the film. Linear sweep voltammetry (LSV) measurements indicate that the designed electrochemical sensor was sensitive and selective and that it achieved a detection limit of 0.10 μM, a limit of quantification of 0.33 μM and a linear range from 0.5 to 15 μM.

**Figure 11:**
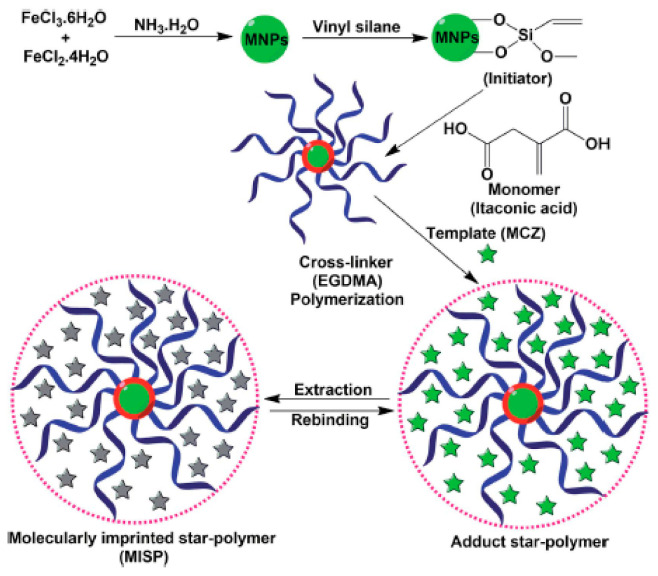
Different steps of fabrication of mancozeb-imprinted star polymer. Reproduced with permission of RSC from [107].

In 2016, Kumar et al. [107] used superparamagnetic iron oxide nanoparticles coated with vinyl silane (silane@SPIONs) and molecularly imprinted star polymers (MISP) to detect and remove Mancozeb (MCZ) from soil and vegetable samples. The authors have used itaconic acid as a functional monomer and EGDMA as crosslinker. An imprinted star polymer synthesis is displayed in Figure 11.

Quantitative measurements, investigated with square wave stripping voltammetry, indicate that the electrochemical sensor has a wide linear range from 5.96 to 257.0 mg·L^−1^ and a detection limit of 0.96 mg·L^−1^. The sensor exhibited excellent selectivity in the presence of different interferents and good stability/reusability after six months of storage.

El-Moghazy et al. [108] developed a sensitive AChE biosensor for pirimiphos-methyl detection in olive oil samples after a simple liquid–liquid extraction. In this study, SPEs were functionalized with electrospun chitosan-polyvinyl alcohol (CS-PVA) blend nanofibrous membranes (NFM), which were activated with glutaraldehyde 1%, prior to incubation with AChE. Inhibition assays were then carried out using pirimiphos-methyl oxon. Amperometric results indicate that the (AChE/CS-PVA NFM/SPE) sensor designed was stable and reproducible during 10 consecutive measurements. The LOD was of the order of 0.2 Nm, corresponding 6 × 10^−5^ ppm.

#### 5.1.3. Pesticide Imprinted Conductive Polymers (PICPs)

**Figure 12:**
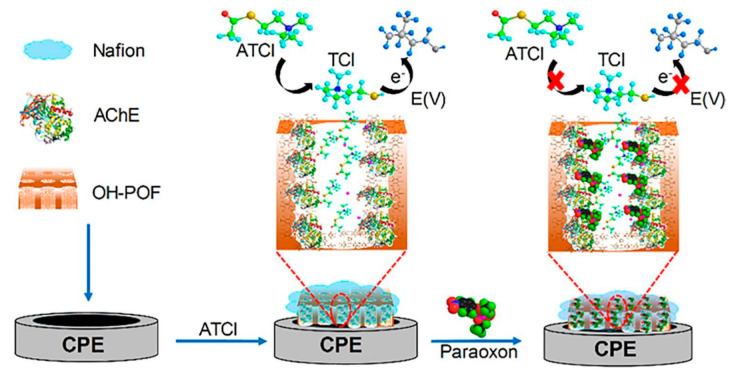
Illustration of the fabrication process of the NF/AChE/OH-POF/CPE biosensor. Reproduced with permission of Elsevier from [109].

Dong et al. [109] reported an electrochemical AChE biosensor based on microporous organic polymers (MOP) for methyl parathion and paraoxon detection in lettuce samples. Herein, phloroglucinol-based MOP was prepared via simple microwave synthesis and then drop coated onto the surface of a carbon paste electrode. AChE was then added and subsequently immobilized by Nafion. The synthesis process and detection procedure are displayed in Figure 12.

DPV electrochemical results were related to the oxidation of thiocholine, produced from ATCI hydrolysis in the presence of AChE. The limits of detection for methyl parathion and paraoxon were of the order of 1.5 × 10^−13^ g·mL^−1^ and 3.4 × 10^−14^ g·mL^−1^, respectively. The linear ranges varied between 5.0 × 10^−13^ to 1.0 × 10^−8^ g·mL^−1^ for methyl parathion and from 1.0 × 10^−13^ to 1.0 × 10^−9^ g·mL^−1^ for paraoxon.

Yassa et al. [110] modified a graphite electrode with thienopyrrole based conjugated poly{1-(5-(4,8-bis(5-(2-ethylhexyl)thiophen-2-yl)benzo{1,2-b:4,5-b’}dithiophen-2-yl)furan-2-yl)-5-(2-ethylhexyl)-3-(furan-2-yl)-4H thieno{3,4-c}pyrrole-4,6(5H)-dione} (PFTBDT) and carbon dots (CDs) for the detection of catechol phenolic compounds used in pesticide synthesis. In this study, PFTBDT was synthesized via Stille polycondensation reaction and then coated on a CD’s modified electrode. This step was followed by the immobilization of laccase enzyme onto the modified electrode, using glutaraldehyde 1% as a cross-linker agent. Several parameters were optimized in this study, such as the amounts of carbon dots, of PFTBDT, of enzyme and the pH of the operating media. Amperometric measurements indicate that the proposed biosensor exhibits an LOD of 1.23 μM, a sensitivity of 737.4 μA·mM^−1^·cm^−2^ and a wide range between 1.25 to 175 μM.

Akdag et al. [111] designed an electrochemical AChE biosensor for paraoxon detection using polypyrrole and chitosan modified platinum (Pt) electrode. The authors electropolymerized pyrrole monomers, by cyclic voltammetry, on platinum electrode (Pt/PPy) and then coated it with a chitosan solution. The chi/Pt/PPy modified electrode was after that incubated in a buffer solution containing glutaraldehyde in order to immobilize the AChE enzyme. The sensing properties of the biosensor were monitored by DPV. LOD was of the order of 0.17 nM, and the sensor exhibited 72% of stability after 60 days.

Kondawar et al. [112] modified a graphite electrode surface with two layers of conducting polymers to design an AChE biosensor for Acephate quantification. In this study, pyrrole monomers were first electrochemically deposited by CV, in the −0.6–0.9 V range for 10 cycles at scan rate 50 mV/s, onto a graphite electrode. Subsequently, aniline with CNTs was electropolymerized by CV on the polypyrrole modified graphite electrode surface, prior to the immobilization of AChE. Chronoamperometry results indicated that the biosensor presents an LOD of 0.007 ppm.

Turan et al. [113] designed a butyrylcholinesterase (BChE) amperometric biosensor for the quantitative determination of paraoxon in milk and tap water. In this work, bis(octyloxy)-di(thieno-thiophen-2-yl)benzooxoadiazole (TTBO) was electropolymerized, by cyclic voltammetry, on a graphite electrode surface prior to the immobilization of silver nanowires (AgNWs) and then BChE. Glutaraldehyde was used to improve the electron transfer, the sensitivity and the selectivity towards paraoxon. The poly(TTBO)/AgNWs/BChE biosensor revealed an LOD 0.212 μM and a sensitivity of 8.076 μA μM^−1^ cm^−2^.

Guler et al. [114] constructed a conducting polymer on a GCE for the quantification of malathion in parsley leaf samples. The working electrode consisted of poly(terthiophene-3-carbaldehyde) (PTT) electrosynthesized on GCE by cyclic voltammetry (in the range from 0.8 to 1.5 V). After that, AChE was immobilized on the PTT film surface and covalently cross-linked by glutaraldehyde. The biosensor response was dependent on the oxidation of thiocholine, which is the hydrolysis compound of acetylthiocholine iodide, catalyzed by AChE activity. CV electrochemical results indicated that the LOD was of the order of 4.08 nM and that the sensitivity and the recovery were equal to 183.2 μA/mM and 97%, respectively.

Bhardwaj et al. [115] designed an immunosensing platform on the basis of a thin film assembly of Cu-MOF (Cu_3_(BTC)_2_@SiO_2_) and 2-amino terephthalic acid (NH_2_-BDC) doped polyaniline (PANI) to detect traces of atrazine. In this study, the conducting film was synthesized by mixing NH_2_−BDC and aniline monomers in an ice bath and then by spin-casting the mixture onto a four electrode sensor surfaces. This step was followed by the spin-casting of Cu_3_(BTC)_2_@SiO_2_ on BDC-PANI electrode surface and by annealing at 100 °C to establish a good bonding. The modified thin film was then bioconjugated with anti-atrazine antibodies. This immunosensor was reproducible and reliable and exhibited an LOD of 0.01 nM.

Salih et al. [116] modified the surface of a carbon paste electrode (CPE) with p-phenylenediamine (p-PD) conducting polymer and ionic liquid (IL) for carbaryl detection in spring water and fruit samples. In this work, different amounts of IL were mixed with graphite and paraffin oil to fabricate IL modified carbon paste electrode (IL/CPE). Two p-PD electropolymerization strategies on IL/CPE surfaces were investigated: (i) by cyclic voltammetry in the range −0.4 to 0.8 V for 40 cycles; and by (ii) potentiostatic mode at a fixed potential 0.7 V during 120 s. Several experimental conditions were tested and optimized: the ionic liquid ratio in paraffin oil, the number of polymerization cycles, pH and the preconcentration duration. DPV results indicated that the poly-pPDs-IL/CPE sensor presents an LOD of 0.09 mmol·L^−1^ and reasonable recovery values between 96 to 117.4%.

#### 5.1.4. Summary of Pesticide Imprinted Polymer-Based Electrochemical Sensors

Table 4 reports characteristics and sensing properties of shortlisted imprinted systems designed for the selective detection of pesticides. Outstanding LODs are reported, i.e., in the sub-nanomolar or in near picomolar regime. Chitosan-based imprinted materials seem to permit reaching extreme LOD values.

### 5.2. Ion Imprinted Polymers

#### 5.2.1. Ion Imprinted Vinylic Polymers (IIVPs)

**Figure 13:**
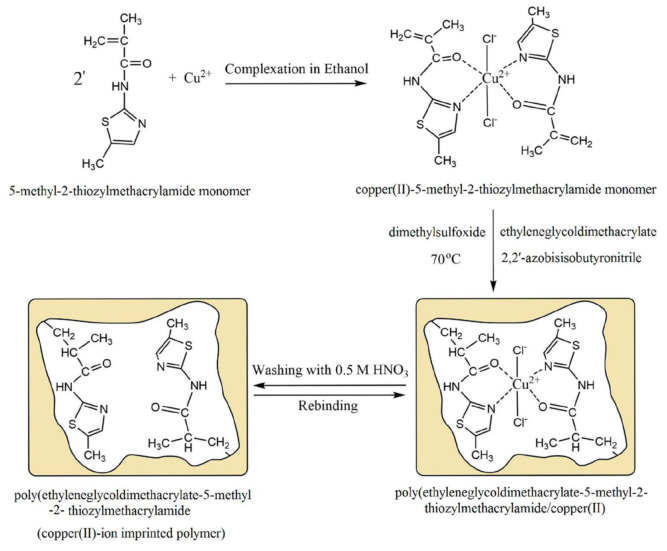
Schematic diagram for the preparation of the copper(II)-ion-imprinted polymer. The acrylamide derivative bearing thiozyl group serves as monomer and ligand in the same time. Adapted with permission of Taylor and Francis from [122].

Organic imprinted polymers are synthesized mostly via free radical polymerization. Basically, vinyl groups are an appropriate category of polymerizable materials for such aim [18]. These polymerizable chelators are known as bifunctional agents; they possess a functionality according to their complexing capability [117]. Moreover, their activity extends to their vinyl function [4]. Crosslinking is an important point either in the presence of one or more functional monomer bearing a ligand [118] or a non-polymerizable ligand such as terpyridine among others [119,120] or with linear chain polymers. This step has been classified into various mechanisms [117]. It comprises crosslinking of linear polymers loaded with metal-binding groups (for example, vinyl pyridine), besides chemical immobilization of commercially [121] or non-commercially available vinylated chelators that may interact with metal ions (see Figure 13 [122]).

**Figure 14:**
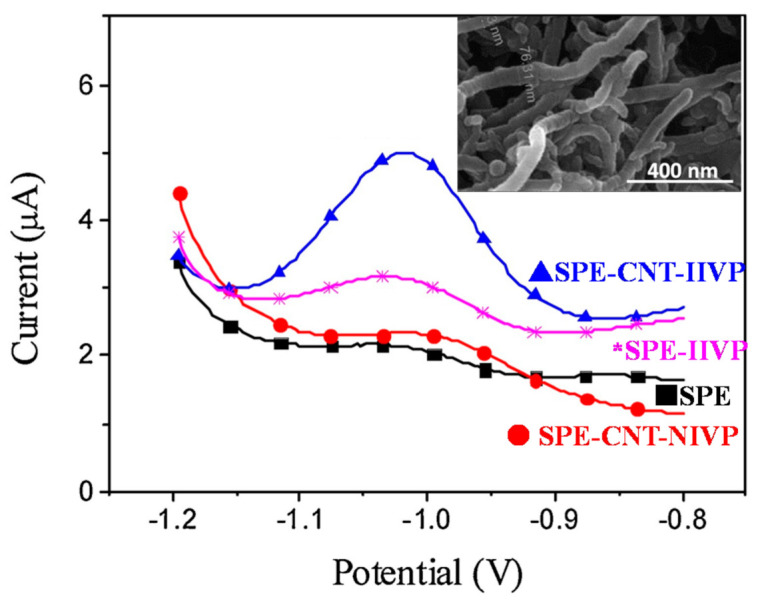
DPV output of 3.0 × 10^−5^ mol L^−1^ Eu^3+^ on bare and differently coated SPE electrodes at pH 4.7. Adapted with permission of Elsevier from [124].

Besides the choice of monomers, free or vinylated ligands, nanostructuration is also important. For example, multiwalled carbon nanotubes (MWCNTs) were used to make MWCNT-IIP nanocomposites for CPEs [123] or dispersed over SPE prior to IIP coating via surface-confined radical photopolymerization [124]. A 4-fold higher response was obtained for the SPE-CNT-IIVP sensor of Eu(III) compared to SPE-IIVP, which is the same system but without any MWCNTs (Figure 14). In this work, surface-confined UV-triggered photopolymerization was conducted with AIBN (Type I photoinitiator) and was found to provide electrochemical sensor with superior performances [124].

In another study, a complex ion imprinted nanocomposite was designed based on both MWCNTs and halloysite nanotubes where the latter were grafted with hyperbranched IIP and the former ensured electron transfer to GCE [125].

#### 5.2.2. Ion Imprinted Conductive Polymers (IICPs)

Electrically conductive polymers are ideal for electrochemical applications because they do not require nanostructuration with carbon or metal to impart conductivity, and could be directly prepared on electrode surfaces within seconds to few minutes. They could also be prepared by precipitation oxidative polymerization in less than 2 h at RT, particularly polypyrrole. However, despite their physicochemical properties, they are only seldom applied for making imprinted polymer-based electrochemical sensors for the selective detection of metal ions. Recently, some of us summarized the findings on polypyrrole-based electrochemical sensors including ion imprinted polypyrroles [68]. Herein, we concentrate on the recent progress in the domain of ion imprinted conductive polymers, mainly polypyrrole, polyaniline and poly(phenylene diamine).

**Figure 15:**
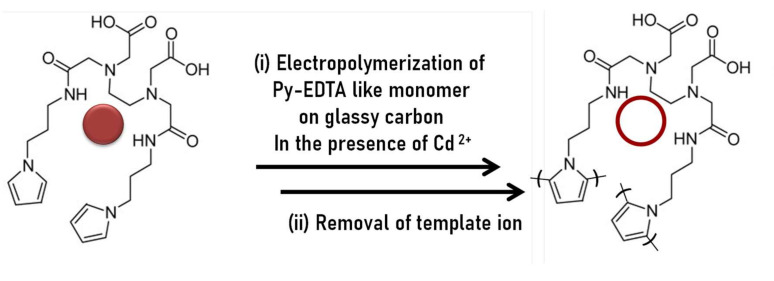
Preparation of Cu(II) imprinted poly(pyrrole-EDTA like) polymer for the selective detection of Cd^2+^. Step (i): preparation of the metallo-polymer by electropolymerization of pyrrole-EDTA like/Cd(II) metal ion complex; step (ii): template ion removal for generating artificial receptor sites within the poly(pyrrole-EDTA like) polymer matrix. Adapted with permission of John Wiley & Sons from [126].

Before we summarize the recent progress on ion imprinted conductive polymers (IICPs), we would like to stress again that, contrary to vinylic polymers, CPs have rigid structure and crosslinkers are quasi never employed. Despite the remarkable progress in the domain of imprinted polymers, little information is available on ion imprinted conjugated polymers for electroanalysis of heavy metal ions. Handpicked examples of recent IICPs are reported in Table 5 including an attractive study of imprinted EDTA-like PPy. In the latter, the teams of Rivas and Moutet explored the propensity of sensors based on PPy/EDTA-like films for selectively detecting Hg(II), Pb(II), Cd(II) and Cu(II) (Figure 15). The Cd(II)-imprinted conductive films were effectively selective towards Cd(II) over other competing metal ions in metal ion mixtures [126].

**Figure 16:**
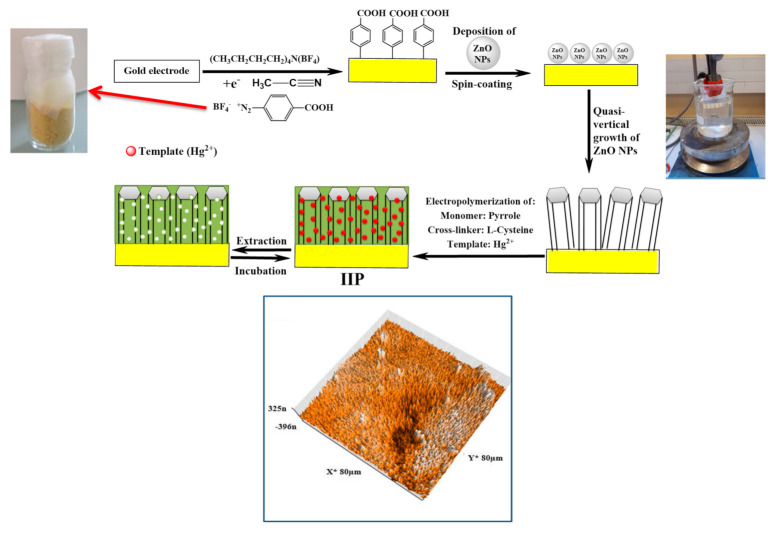
Top: Schmatic illustration of the stepwise synthesis of mercury imprinted PPy wrapped around vertically aligned ZnO nanorods attached to diazonium-modified gold electrodes. Bottom: (80 × 80 μm^2^) 3D image of Au-diazo-ZnO NRs. Reproduced from [14].

Whilst pyrrole derivative bearing ligands are interesting, they might request synthesis efforts as in the case of chelatant-bearing vinylic monomers. For this reason, pyrrole can be polymerized in the presence of metal ions and ligands/chelators. Some used, in this regard, L-cystein and acrylic acid which served both as co-dopants and ligands [127]. The resulting ion imprinted polymer permitted to achieve picomolar LOD for a lead as reported by Ait-Touchente et al. [14]. This is the lowest LOD ever reported for Hg(II) detection. Figure 16 schematically illustrates the making of nanostructured Hg(II) ion imprinted polypyrrole coated on ZnO nanorods that were vertically aligned on arylated gold electrodes.

#### 5.2.3. Ion Imprinted Sol-Gels (IISGs)

Silanes are mainly used in sol-gel polymerization in order to obtain gels, coatings and particles, all suitably adapted for the fabrication of IIP-based electrochemical sensors. A few processes have been shortlisted and summarized hereafter.

##### Detection of Copper Ions Cu(II)

**Figure 17:**
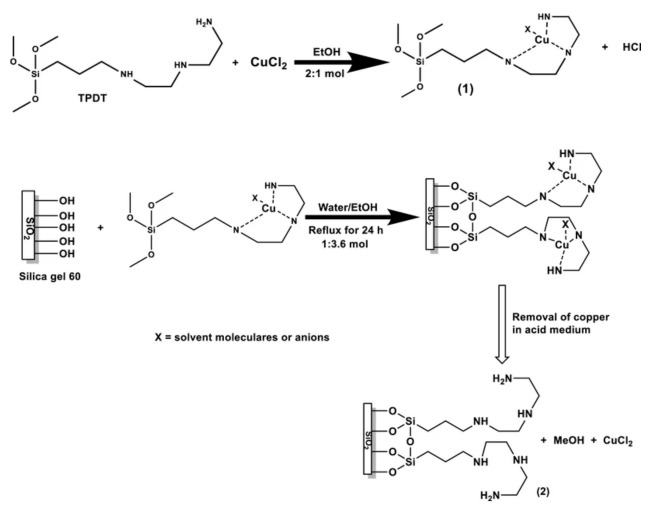
Synthesis of copper imprinted TPDT-functionalized silica. Reproduced with permission of Elsevier from [128].

Copper ions were detected using copper imprinted sol-gel [128] bearing N1-(3-(trimethoxysilyl)propyl)diethylenetriamine (TPDT), which has the property of complexing copper ions with its diethylenetriamine group. The first step of IIP synthesis was the synthesis of ligand-functionalized silane. After complexation of Cu(II) for 24 h, the gel was crosslinked at reflux and washed to leach the Cu(II). This resulted in a copper ion imprinted sol-gel material (Figure 17) that served for making carbon paste electrode to track Cu(II) in tap water. LOD and sensitivity depended on pre-concentration time, optimally set at 1800 s. If LOD remains in the sub-micromolar regime, it is interesting to note that this IISG did not require any crosslinker such as TEOS and the synthesized TPDT silane was sufficient to make a 3D imprinted network. Interference studies have been done with Fe(II), Zn(II), Pb(II) and Ni(II). Another feature was the selectivity of the IISG to copper over Ni(II), Zn(II) and Pb(II).

##### Detection of Cadmium Ions Cd(II)

**Figure 18:**
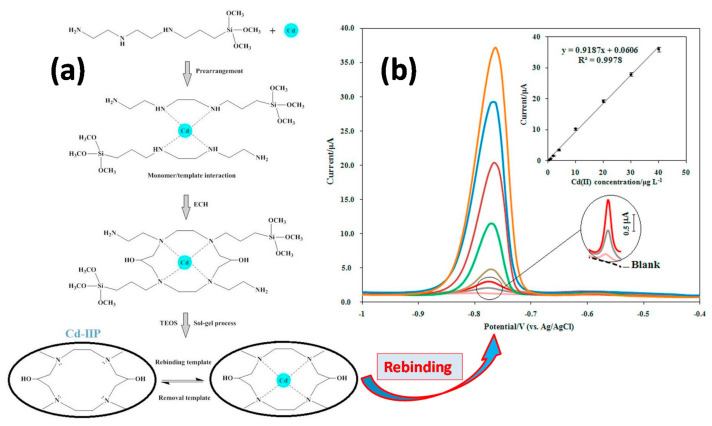
Synthesis of a cadmium ion imprinted sol-gel (**a**), and the use of its corresponding carbon paste for the highly sensitive detection of Cd(II). (**b**) Square wave voltammograms of Cu(II) detection and its further calibration curve. Reproduced with permission of Elsevier from [129].

In a similar study, CPE was prepared with cadmium imprinted sol-gel [129]. 3-(2-(2-aminoethylamino)ethylamino)propyl-tri methoxysilane (AAAPTS) was used as functional monomer, epichlorohydrin as a cross-linker and Cd(II) ions as a template; TEOS was used for the sol-gel process (Figure 18). The reaction of epichlorhydrin with the NH groups opens the epoxy ring and yields OH groups which enhance the hydrophilic character of the imprinted gel. After washing off Cd(II) with HCl, the final IIP was obtained as fine powder and mixed with carbon. CPE was employed to track Cd(II) in aqueous solutions prepared in lab, and in environmental water samples. The IISP had an LOD of 0.15 μg Cd.L^−1^, the linear range was 0.5–40 μg·L^−1^ and exhibited outstanding selectivity despite 30 to 100 fold more concentrated competitive metal ions. Indeed, no loss in recovery of Cd(II) was noted in the presence of other ions.

##### Detection of UO_2_^2+^

**Figure 19:**
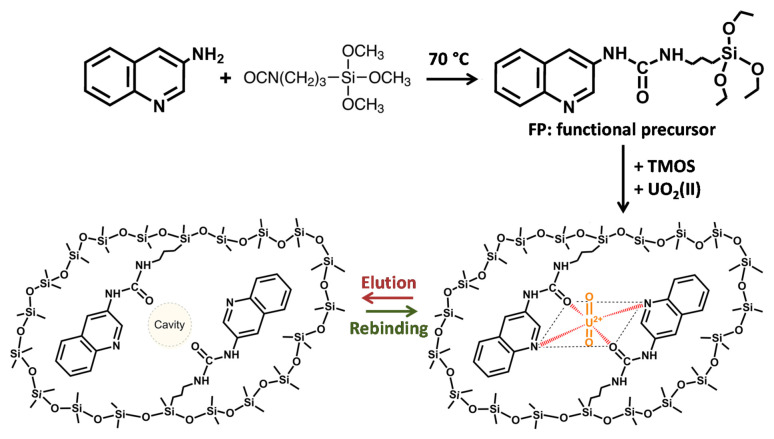
Synthesis of IISG from quinolone-functionalized silane, TMOS and uranyl. Adapted with permission of Elsevier from [130].

An interesting IISG has been proposed by Güney and Güney [130] with rarely employed 3-isocyanatopropyl trimethoxysilane (ICTMS) that was reacted with 3-aminoquinoline in order to obtain a functional silylated monomer bearing a quinolone ligand (Figure 19). The latter was crosslinked using tetramethylorthoxysilicate (TMOS) in the presence of UO_2_(II). A CPE was made by mixing the UO_2_(II)-imprinted sol-gel with carbon powder. The CPE exhibited an LOD of 3.07 × 10^−10^ mol·L^−1^; the linear range was 2.0 × 10^−9^–3.0 × 10^−7^ mol·L^−1^. The sensor could be used to selectively detect uranyl in tap, pond and waste waters, with good recovery.

##### Detection of Europium Eu^3+^

Europium is a reactive rare earth. The increasing applications of this element in the domain of industrial applications, material science, electronic engineering and life science raised toxilogy concerns, hence its traceability using IIPs [131].

**Figure 20:**
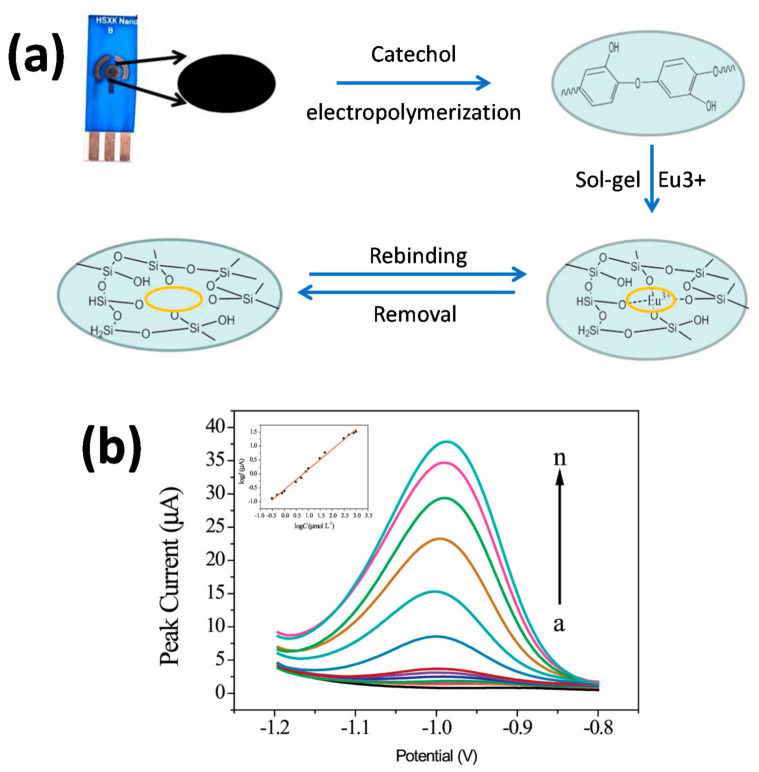
Two step preparation (**a**) and electrochemical Eu(III) sensing performance (**b**) of screen printed electrode coated with polycatechol-IISG bilayer. Adapted with permission of Elsevier from [131].

The working electrode consisted of an SPE, coated with electrosynthesized polycatechol (PC), a signal amplifying element, and an IISG (Figure 20). The signal-amplifying element PC bears hydroxyl groups and oxygen which could coordinate Eu^3+^, therefore improving the sensitivity and selectivity of Eu^3+^ IISG. The ion-imprinted sol-gel solution was prepared using TEOS, phenyltrimethoxysilane (PTMOS), methyltrimethoxysilane (MTMOS) and Eu^3+^ solution. Note, however, that no reason was found to justify PTMOS and MTMOS silanes besides the TEOS crosslinker.

The characterization of the fabricated electrode was performed by CV and EIS. Results show clear differences before and after Eu^3+^ removal: the current is weak and the resistance is strong before removal; after removal, a redox peak on CV was noted, and the resistance decreased significantly. A clear difference between the IIP with and without PC has been observed: the peak current is nearly twice as strong for the IIP with PC, hence the effective signal amplifying property of PC.

DPASV has been used to determine the LOD and the linear range, and they are respectively 1.0 × 10^−7^ mol·L^−1^ and 0.3–1000 μmol·L^−1^. The sensor was found to be selective to Eu^3+^ over other metal ions. Indeed, peak current did not show any significant changes with the presence of competitive ions such as Ni^2+^, Co^2+^, Cu^2+^, Fe^3+^ or Gd^3+^.

The detection of Eu(III) was achieved using a bilayer of organic polymer that facilitates electron transfer and a sol-gel imprinted polymer that facilitates selective recognition. Recently, an ion imprinted hybrid polymer system (IIHP) has just been described, and consisted of imprinted, crosslinked vinylic polymers and sol-gel. –SH from MPS and the imidazole group from the vinylic functional monomer have a synergetic effect of Cd(II) complexation [132]. Each system (organic or sol-gel) had its own functional and crosslinker monomers (see Table 5), but 3-(trimethoxysilyl)propyl methacrylate served as a coupling reagent for organic and inorganic phases. Indeed, it is a bifunctional molecule enabling involvement in radical polymerization via the methacrylate end, and involved in sol-gel synthesis via the the trimethoxysilyl part. This sensor is certainly robust and highly selective; however, DPASV did not show any striking difference between the carbon paste electrode prepared from graphite powder only, and those prepared with IIHP and NIHP. The decreasing trend of the current intensity was CPE-IIHP (100 µA) > CPE-NIHP (80 µA) > CPE (55 µA).

#### 5.2.4. Summary of Experimental Conditions of Preparation and Performances of Ion Imprinted Polymers

Table 5 summarizes the experimental conditions for the synthesis of ion imprinted polymers as thin films or nanocomposites. An organic medium is required for vinylic polymers, whilst water/alcohol usually is considered for sol-gels. Conductive polymers require aqueous media, which is interesting in this respect, making the process “greener” and energy saving since the synthesis is usually conducted at RT. There is no clear trend related to electrosensing; DPV and SWV seem to be randomly employed and return excellent LODs. From the shortlisted case studies, obviously CPEs are the most investigated electrodes and one should expect their tremendous development in the near future by “Imprinters”.

### 5.3. Bacteria Imprinted Polymers

Pathogens are infectious microorganisms, harmful to humans. This section will emphasize waterborne pathogens. There are several ways to prepare MIP-based electrochemical sensors based on whole cell imprinting, surface imprinting, bacterial protein imprinting, quorum signaling molecules, spores or molecules that reflect the activity of the bacteria. The various approaches will be discussed through handpicked case studies.

#### 5.3.1. Whole Cell Imprinting

**Figure 21:**
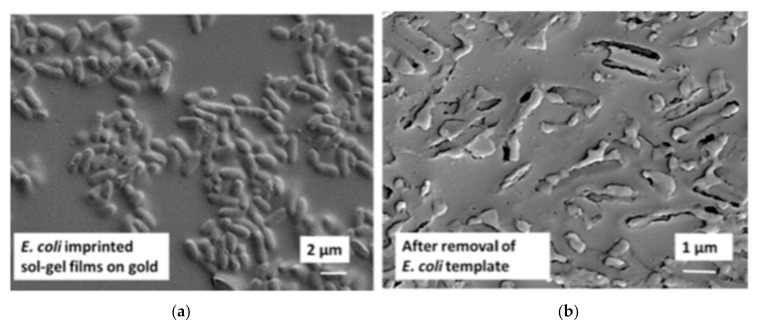
SEM images of *E.coli* imprinting before (**a**) and after removal (**b**) of templates. Reproduced with permission of Elsevier from [137].

*Escherichia coli* (*E. coli*) is a rod shaped bacterium; it is normally harmless to humans and can generally be found in their intestines. However, a few varieties can cause diseases such as abdominal cramps, bloody diarrhea and vomiting. These can be found in contaminated water or food. Jafari et al. [137] proposed an electrochemical sensor in which the sensing layer is a polymer layer synthetized by sol-gel method; bacteria are added to the fresh sol solution at the end of the process. Tetraethoxysilane (TEOS) has been used as the monomer for the polymerization; it also serves as a crosslinker. EIS measurements were done with the following parameters: amplitude of 10 mV at open circuit potential with a frequency range of 100 kHz–0.1 Hz. Results showed good selectivity when the recognition of *E. coli* and *S. aureus* (a spherical bacteria) was compared: when the sensor captures the corresponding bacteria, the charge transfer resistance increases; in the case of *S. aureus*, the change of signal is greatly reduced. The same is true for *Pseudomonas aeruginosa*, another rod shaped bacterium. The performance of impedimetric sensor for *E.coli* is lower than other impedimetric sensor using anti-*E. coli* antibodies, but it has a low cost and low LOD. The authors have chosen the whole cell imprinting technique. This raises the issue of bacteria extraction from the in situ synthesized MIP film; as one can see in Figure 21, the washing of imprinted bacteria seems to seriously damage the imprinted sites.

#### 5.3.2. Bacterial Surface Imprinting

**Figure 22:**
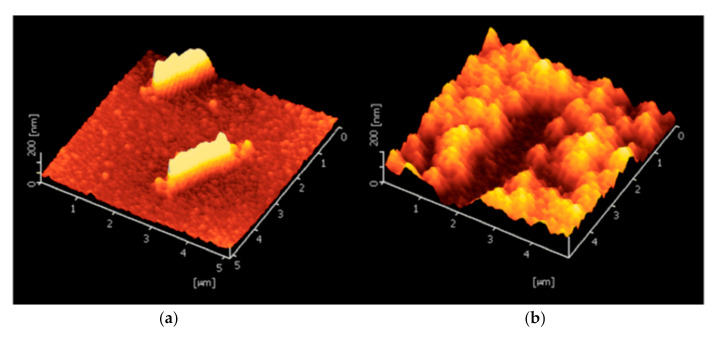
AFM image of bacteria imprinted polymer, before (**a**) and after (**b**) washing. Reproduced with permission of Elsevier from [63].

As noted from the SEM picture above (Figure 21b), the imprinting of whole bacteria is not well adapted due to the size of the template; however, the imprinting of the surface or of a portion of bacteria is possible and a better option (Figure 22). Sulfate reducing bacteria (SRB) are anaerobic microorganisms naturally present in environmental sources such as soil, sea or river. They obtain their energy by reducing sulfate to sulfite, which is a highly corrosive and toxic substance. An imprinted chitosan-based electrochemical sensor has been fabricated in this regard by Qi et al. [63]. Chitosan (CS) becomes insoluble if the pH of the solution is higher than 6.3. In order to coat this biopolymer on the electrode surface, a potential was applied on the cathode in order to reduce H^+^ to H_2_, thus making the pH reach the threshold for deposition. Then, SRB bacteria were coated on the surface of the biopolymer film; only a part of the surface of SRB was imprinted. However, the washing of bacteria after imprinting can induce enlargement or deformation of the prints (the recognition sites). The impedimetric measurements show very good selectivity for SRB over the other *S. aureus*, *M. luteus*, *V. alginolyticus* and *V. anguillarum* bacteria as demonstrated in Figure 22.

**Figure 23:**
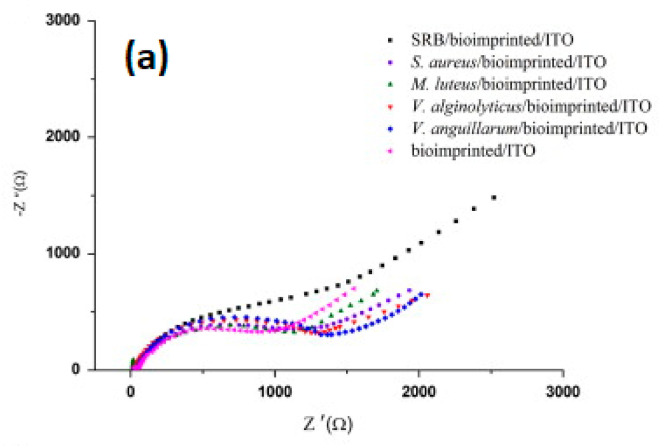

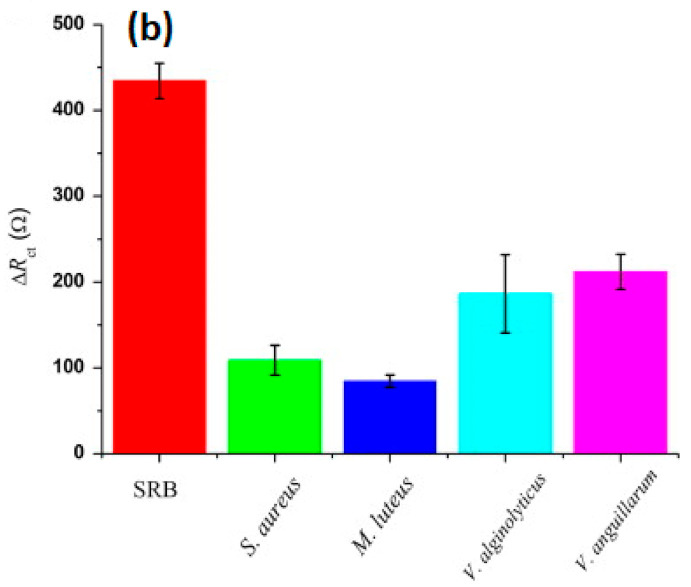
Impedance spectra obtained with bioimprinted sensor and the biosensor after incubation with 1.0 × 10^8^ cfu mL^−1^ SRB, S. aureus, *M. luteus*, *V. anguillarum*, and *V. alginolyticus* in PBS containing 5 mM Fe(CN)_6_^4−/3−^ as the probe (**a**).The comparison of Rct changes of the impedimetric biosensor based on SRB-mediated bioimprinted film to SRB, *S. aureus*, *M. luteus*, *V. anguillarum*, and *V. alginolyticus* (**b**). DRct is the change of charge transfer resistance of impedimetric sensor before and after incubation with different bacteria. Reproduced with permission of Elsevier from [63].

Figure 23 shows Nyquist plots for bioimprinted ITO before and after attachment of several bacteria (Figure 23a). As the receptor sites were shaped by SRB, this bacterium is recognized most. The Nyquist plot, for bioemprinted ITO with adsorbed SRB, shows the largest semi-circle on the Z’ axis. The difference between Z’ values for ITO, with and without bacteria, indicates resistance to charge transfer (ΔR_ct_). It is the largest value for SRB because the sensor is indeed selective towards this bacterium (Figure 23b).

This type of sensor is based on the size and shape of the bacteria, but the recognition site is fragile; a study on its reusability would be interesting, as one knows that biological bio-recognition elements, for example antibodies, can easily be damaged.

#### 5.3.3. Bacterial Protein A Surface Imprinting

Imprinting a part of the surface of a pathogen is more efficient than imprinting a whole cell; however, the target analyte is still too massive to enable recognition by functional groups. This disadvantage can be bypassed if the target analyte is not the whole bacterium, but its corresponding molecules and proteins—for example, a specific surface protein. In this regard, Khan et al. [138] employed protein A as a template protein for making an imprinted polymer for the recognition of *Staphylococcus aureus* (*S. aureus*). 3-aminophenol was electropolymerized by CV on a film of single walled carbon nanotubes (SWNCTs) on which a solution of protein A has been deposited. Removal of a protein A (PA) template was done using proteinase K, an enzyme that naturally degrades the former. The fabricated sensor shows good sensitivity when an EIS test with PA and bovine serum albumin (BSA) has been done; however, testing with the surface protein from other bacteria would have given more credit to this work and the efficacy of the sensor in selectively recognizing *S. aureus*.

#### 5.3.4. Imprinting of Bacterial Flagella Proteins

**Figure 24:**
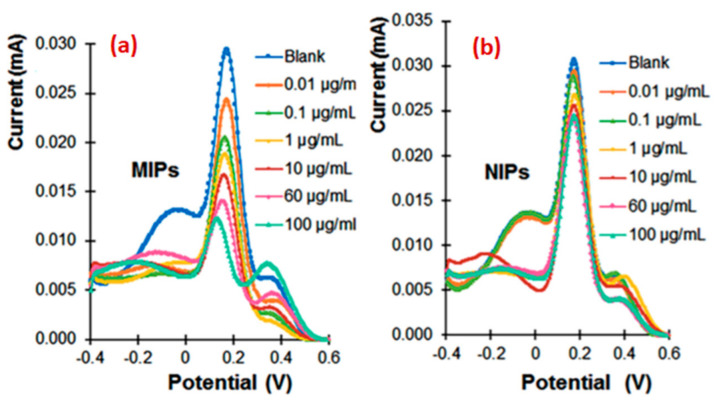
SWV voltammogram: result for MIP with different concentration of flagella ((**a**), left) and result for NIP ((**b**), right). Reproduced with permission of Elsevier from [139].

Another strategy is to detect proteins from the flagella of bacteria. This approach is adopted by Khan et al. [139] for the detection of *Proteus mirabilis*. This bacterium can infect the respiratory tract, urinary tract and open wounds, causing fever and pain. Its flagella permits *P. mirabilis* to move in biological environments; more importantly, they are specific and thus a means for the identification of this bacterium. Phenol was used as a monomer and electropolymerized by CV (range from −0.2 V to 0.8 V; scan rate 50 mV/s; 15 cycles). The methods of detection are EIS and SWV; results show an excellent response for each method: for EIS, the impedance increases the more the sensing layer captures target molecules. For SWV, the pic current decreases when the concentration of flagella increases (Figure 24a). However, no such changes in the sensor response were noted with the non-imprinted polymer (Figure 24b) The selectivity is also good when the sensor was used for a mixture of flagella/BSA and flagella/PA, and the percentage of deviation in the response caused by interference is small for EIS (<8%) and SVW (<5%). The use of two methods of electrochemical detection enables crosschecking the results and adopting the most suitable strategy of protein detection.

#### 5.3.5. Bacterial Spore Imprinting

Spores are increasingly investigated for bacteria identification as they are generally released when these latter are under stress. Lahcen et al. [140] have functionalized surfaces of carbon paste electrodes with polypyrrole imprinted polymer for *Bacillus cereus* spore detection, used as simulant for *Bacillus anthracis* spores. Polypyrrole films were electropolymerized by cyclic votammetry (5 scans between −0.7 V and +0.7 V at 100 mV/s), prior to the addition of 10^4^ CFU/mL bacterial spores and further electropolymerization for 5 cycles at 100 mV/s. Spores were then removed by sonication for 5 min in distilled water or by incubation in a surfactant.

Several parameters were investigated and optimized, mainly the monomers’ concentration, the number of scans, the nature of the extractor, the incubation time and the sonication duration. The designed sensor exhibits a good selectivity towards *Bacillus cereus* spores and a dynamic range ranging from 10^2^ to 10^5^ CFU/mL, which makes it suitable for effective measurements of *Bacillus cereus* spores.

#### 5.3.6. Imprinting Quorum Sensing Signaling Molecules

**Figure 25:**
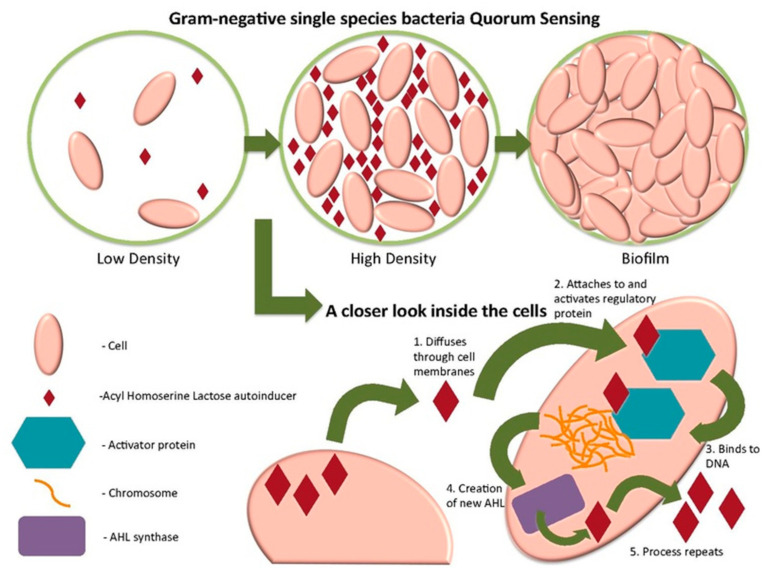
Mechanisms of quorum sensing from isolated bacteria to the formation of biofilms. Early detection of quorum sensing signaling molecules will require action to prevent biofilm formation. https://www.wikiwand.com/en/Quorum_sensing; last accessed 8 June 2021.

For the detection of the bacterium *Aeromonas hydrophila*, Jiang et al. [141] chose to imprint N-acryl-homoserine-lactones (AHLs) molecules that can induce the expression of pathogenic factors. AHLs participate in the quorum sensing system (QS), a system that enables communication between bacteria, their gathering and biofilm formation. Quorum sensing plays a key role in determining virulence (Figure 25).

AHL is generally produced at a low concentration; therefore, it is difficult to detect. MIP technology can thus solve this problem owing to the combined high selectivity of MIPs and outstanding sensitivity of electrochemical devices. In this publication, magnetic molecularly imprinted polymers (MMIP) were used as the sensing element. First, Fe_3_O_4_ magnetic nanoparticles were prepared using a solvothermal method; then, silica-shell was prepared using TEOS. Subsequently, 3-methacryloxypropyltrimethoxylane (APTES) was used in order to introduce amino groups. Finally, polymerization was performed at the surface of the magnetic nanoparticles with an analogue template protein (DMHF). The detection of AHLs was done as follows: first, MMIP was incubated in a solution of target proteins, after four minutes, magnetic GCE, the working electrode, is introduced in the solution, and the MMIP will attach magnetically to the electrode. When all detection sites of MMIP are occupied, the electron-transfer resistance between the heart of MMIP and the electrode is maximal. This resistance decreases when captured proteins are washed off from MMIP.

DPV was the sensing technique; results show that the selectivity of the MMIP was good when tests with DMHF in the presence of structural analogues of AHLs including C4-AHL, C6-AHL, C8-AHL and N-3oxo-C6-HSL were conducted. The electrochemical sensor was found to be stable: a test was conducted with MMIP stored for about three months; no particular changes were noted. Finally, the detection range of AHLs was found to be in the 2.5 × 10^−9^ mol·L^−1^ to 1.0 × 10^−7^ mol·L^−1^ range

#### 5.3.7. Summary of Bacteria Imprinted Polymers

Table 6 summarizes shortlisted case studies tackled in this mini-review. It concerns the bacterium under test, the monomer employed to make the MIP, the electrode material, the polymerization technique, the electrochemical technique and the limit of detection. Despite the low number of entries, Table 6 testifies for the rich literature on bacterial sensing using MIP-based electrodes and particularly the numerous strategies for recognizing bacteria without necessarily going through the problematic whole cell imprinting technique.

## 6. Conclusions and Outlook

In this review, we have summarized the recent developments of imprinted polymer-based electrochemical sensors (focus on 2017–2021). We have considered vinylic, conjugated and sol-gel type polymers. We targeted pesticides, heavy metal ions and bacteria as templates for the making of imprinted organic and inorganic polymers. From the synoptic tables, thermally induced radical polymerization is time-consuming and requires 24 h; surface-confined photopolymerization is faster. Sol-gel polymerization requires one day to complete, but the polymerization of conjugated polymers is probably the most time-saving technique, particularly when it is electrochemically triggered (a few minutes will suffice). As far as performances are concerned, electrochemistry is an excellent technique to achieve outstanding limits of detection, in the nanomolar or even better, in the picomolar regime. Improvements are certainly brought by new technologies enabling to record signals with high S/N ratios, at extremely low concentrations of analytes. We also discussed the interest of nanostructuration with carbon nanotubes or graphene, but also hybrid filling consisting of nanostructuration with both MWCNTs and clay nanotubes. If direct coating of the imprinted polymers on the electrodes remains a very well established method, the design of nanocomposites prior to their deposition on flat glassy carbon electrodes or their mixture with graphite powder to make carbon paste electrodes seems approved by the Imprinters community and became trendy. Indeed, we witness more and more research studies on imprinted polymer nanocomposites coated on GCE and protected by Nafion or in the form of CPEs.

Achievement of very high performances is tedious, and hundreds of inspiring strategies are offered to the specialist or the newcomers in the field. Protocols should, however, be tested and adapted to the context of the study and to the targeted pollutant be it organic/inorganic compound or pathogenic micro-organisms.

To sum up, the prevailing design of MIP sensors in the last decade has attempted to demonstrate high performances depending on theoretical and functional models. However, in spite of these positive results, more attention is required to improve the MIP sensors synthesis technologies in order to use them in real samples and environment monitoring of pollutants, although much has been done in this sense as noticed in the synoptic Table 4, Table 5 and Table 6.

Note that care should be taken when designing MIP-based electrochemical sensors as reproducibility is an issue. It takes time and several trials/errors to obtain a system that is validated by various labs. This should be done in an inter-laboratory experience to test a given type of electrochemical sensor of waterborne pollutants.

The recovery should not be a concern, and one could clearly note development of low-cost disposable electrodes. Probably in this sense, the development of highly selective and sensitive paper electrodes will avoid the problem of contamination by hazardous compounds as they can be disposed of by simple burning [142].

With future developments, MIP-based electrochemical sensors could reach a high technology readiness level [42] and become alternatives for existing commercially available devices. Molecular imprinting indeed remains a very interesting, viable technology that will possibly be competitive within the (biomedical) diagnostic market in the upcoming years [143].

## Figures and Tables

**Table 1:** Handpicked reviews on MIPs for the detection of chemicals and pathogenic microorganisms.

Running Title	Scope of Review	Year of Publication	Refs.
Monitoring of metals using IIP ^a^s	Overview of IIP fabrication and applications in different domains.	2015	[18]
MIP for electrochemical detection of drugs	This paper critically reviews applications of MIP-based electrochemical sensors for the detection of drugs.	2018	[19]
MIP-based sensor for detection of food hazard	General overview of MIP-based optical, electrochemical and gravimetric sensors of hazardous compounds in food.	2019	[9]
Electrochemical sensors based on MIP and nanomaterials	Recent advances on MIP- and nanomaterial-based electrochemical sensors, without specific targets.	2019	[20]
Overview of recent nanostructured MIP based sensors for pesticide detection	A study on existing NP ^b^-MIP ^b^ based sensors for pesticide, showing their fabrication method and experimental result.	2020	[21]
Applications of chitosan in molecularly and ion imprinted polymers	A brief overview of recent applications of chitosan-based MIPs and MIP composites.	2020	[22]
MIPs—towards electrochemical sensors and electronic tongues	The paper discusses the combination of chemometrics and MIP technology in view of developing electronic tongues	2021	[23]

^a^ IIPs: ion imprinted polymers; ^b^ NP: nanoparticle.

**Table 2:** Selected, commercially available monomers frequently employed for making MIPs. Pyrrole is displayed with its corresponding 2D microstructure.

Functional Monomers		Crosslinkers	
Vinylic Monomers			
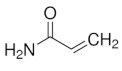	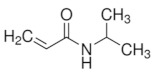	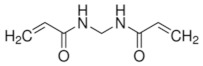	
Acrylamide	*N* -Isopropylacrylamide	*N* -Isopropylacrylamide	
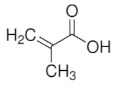	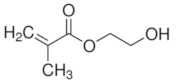	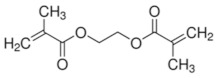	
Methacrylic acid	2-Hydroxyethyl methacrylate	Ethylene glycol dimethacrylate	
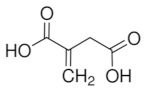		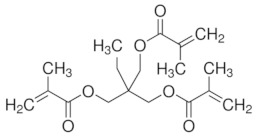	
Itaconic acid		Trimethylolpropane trimethacrylate	
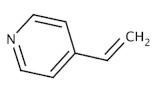	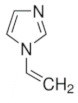	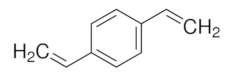	
4-Vinylpyridine	1-Vinylimidazole	*p*-Divinylbenzene	
**Conjugated Monomers**			
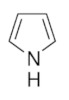	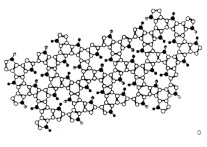	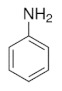	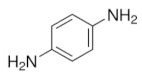
Pyrrole	2D PPy microstructure	Aniline	*p*-Phenylenediamine
**Silanes**			
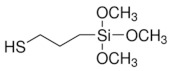	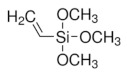	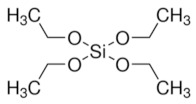 Tetraethyl orthosilicate	
(3-Mercaptopropyl) trimethoxysilane	Vinyltrimethoxysilane		
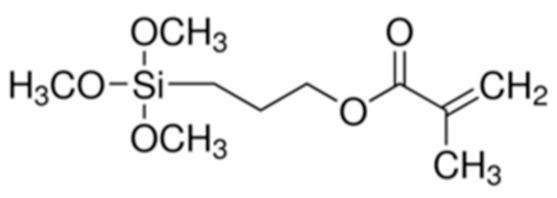			
3-(Trimethoxysilyl)propyl methacrylate			

**Table 3:** Principles, features and applications of electrochemical techniques used in MIP-based electrochemical sensors.

Electrochemical Technique	Principals	General Features and Applications
Cyclic voltammetry	Current measurement as a function of the linear applied potential	- CV provides essential redox processes and information concerning the analysis (matrix, analyte, and electrode). - Not very useful for quantitative determinations.
Differential Pulse Voltammetry	Current measurement between increased pulses of potential with equal increments.	- A low capacitive current which leads to the enhancement of the sensitivity. - Very low and competitive LOD ^a^ values. - Usually applied in the case of irreversible systems or in systems presenting slow-reaction kinetics
Square Wave Voltammetry	Current is determined when an increasing square wave potential is applied.	- Low capacitive current which leads the enhancement of the sensitivity. - Very low and competitive LOD values. - Often applied for the study of reversible or rapid reaction kinetics systems
Amperometric techniques/Chronoamperometry	The application of a constant potential induces the appearance of a corresponding current	- Very useful for continuous monitoring. - Suited to miniaturization and portability. - Difficulty to sense the existence of multiple target analytes in the media.
Stripping voltammetry	A step of analyte pre-concentration precedes its stripping by scan potential application	- Very powerful technique for trace metals and some complexing neutral species determination.> - Requires many optimisation steps.
Electrochemical impedance spectroscopy	Small sinusoidal voltage is applied and complex impedance is measured at the electrode/electrolyte interface	- High sensitivity and specificity - Numerous applications - Non-specific adsorption onto the electrode surface. - Often requiring a Faraday cage to reduce noise. - Theoretical simulation is required for data analysis

^a^ LOD: limit of detection.

**Table 4:** Performances of handpicked pesticide electrochemical sensors.

Pesticide	Sensing Material	Method of Detection	Detection Media	LOD, Sensitivity, Detection Range (DR)	Refs
Hydrazine	ZnO, NF	CV	Tap, sea, and mineral water	LOD = 5 μM S = 0.14 μA·μM^−1^·cm^−2^	[85]
Dichlorvos	TiO_2_/CS	CV, DPV	Cabbage juice	LOD = 0.23 nM DR = 1.13 nM to 22.6 μM	[89]
Dichlorvos	TiO_2_/CS	DPV	Cabbage juice	LOD = 29 nM DR = 0.036 μM to 22.6 μM	[90]
Dichlorvos	TiO_2_/CS	DPV	Juice samples	LOD = 7.4 nM	[91]
Carbamate	MPS	CV	Fruit samples	LOD = 1 nM S = 32.0 μA·cm^−2^.M^−1^	[92]
Fipronil	-	CV	Spiked water samples	LOD = 34 × 10^−5^ μM	[93]
Fenitrothion	GdM	DPV	Soil and water samples	LOD = 5 nM S = 1.36 μA·μM^−1^ cm^−2^	[94]
Trichlorfon	TiO2/CMCS	CV, DPV	Food	LOD = 4 × 10^−7^ M S = 0.5077 µA·M^−1^	[95]
Cypermethrin	MMA (FM), EDGMA (CL), AIBN (In)	CV	Vegetable juice	LOD = 15 ppb S = 0.094 μA·ppm^−1^	[97]
Cypermethrin	CHAC, resorcinol, dopamine	CV	Crayfish, squid, soil and water	LOD = 6.7 × 10^−14^ M	[98]
Glyphosate	CS	EIS, CV	River water	LOD = 0.001 pg/mL	[99]
Glyphosate	PPy	SWV	Spiked water samples	LOD = 1 pM	[13]
Malathion	Bisacrylamide, TMEDA, APS	EIS, CV, DPV	Olive oil and fruit samples	LOD = 0.06 pg·mL^−1^	[100]
Methyl parathion	MAA (FM), EGDMA (CL), AIBN (In)	-	Fish samples	LOD = 1.22 × 10^−6^ mg·L^−1^	[101]
Methyl parathion	quercetin, resorcinol	CV	Water, fruit and vegetable juice	LOD = 0.01 μM	[102]
Methyl parathion	Zinc porphyrin, EGDMA (CL), AIBN (In)	DPV	Apple samples	LOD = 31.6 nM	[103]
Phosalone	-	SWV	Fruit, lake water, and soil	LOD = 0.078 nM	[104]
Profenofos	SiO_2_-vinylcarboxylat	-	Vegetable samples	LOD = 2 nM S = 0.573 A·M^−1^	[105]
Imidacloprid	VBA, EGDMA (CL)	LSV	Brown rice samples	LOD = 0.10 μM	[106]
Mancozeb	IA (FN), EGDMA (CL)	SWV	Soil and vegetable samples	LOD = 0.96 mg·L^−1^ DR = 5.96–257 mg·L^−1^	[107]
Pirimiphos-methyl	CS-PVA, Gl, PMO	-	Olive oil	LOD = 0.2 nm	[108]
Methyl parathion	Phloroglucinol, NF	DPV	Lettuce	LOD = 1.5 × 10^−13^ g·mL^−1^ DR = 5 × 10^−13^ to 1.0 × 10^−8^ g·mL^−1^	[109]
Paraoxon	Phloroglucinol, NF	DPV	Lettuce	LOD = 3.4 × 10^−14^ g·mL^−1^ DR = 1.0 × 10^−13^ to 1.0 × 10^−9^ g·mL^−1^	[109]
Catechol	Thienopyrrole, PFTBDT, Gl	-	Tap water	LOD = 1.23 μM S = 737.4 μA·mM^−1^·cm^−2^ DR = 1.25 to 175 μM	[110]
Paraoxon	PPy, CS	DPV	Spiked water samples	LOD = 0.17 nM	[111]
Acephate	PPy, aniline	CA	Spiked water samples	LOD = 0.007 ppm	[112]
Paraoxon	TTBO, Gl	-	Milk and tap water	LOD = 0.212 μM S = 8.076 μA μM^−1^ cm^−2^	[113]
Malathion	PTT	CV	Parsley leaves samples	LOD = 4.08 nM S = 183.19 μA/mM	[114]
Atrazine	NH_2_-BDC, PANI	-	Spiked water samples	LOD = 0.01 nM	[115]
Carbaryl	p-PD, IL	DPV	Spring water and fruit	LOD = 0.09 mmol·L^−1^	[116]

FM = functional monomer, CL = cross-linker, In = initiator, CV = Cyclic voltammetry, EIS = electrochemical impedance spectroscopy, DPV = Differential pulse voltammetry, CA = Chronoamperometry, SWV = Squarewave voltammetry, NF = Nafion, CS = chitosan, MPS = 3-mercaptopropyl)-trimethoxysilane, GdM = gadolinium molybdate (Gd_2_MoO_6_), CMCS = Carboxymethyl chitosan, MMA = methyl methacrylate, EGDMA = ethylene glycol dimethacrylate, AIBN = 2,2′ azobis(2-methylpropronitrile), CHAC = activated carbon prepared from coconut husk, TMEDA = N, N, N, N-tetramethyl ethylenediamine, APS = ammonium persulfate, MAA = methacrylic acid, VBA = p-vinylbenzoic acid, IA = itaconic acid, PVA = polyvinyl alcohol, PMO = pirimiphos-methyl oxon, Gl = glutaraldehyde, polypyrrole, PFTBDT = 1-(5-(4,8-bis(5-(2-ethylhexyl)thiophen-2-yl)benzo{1,2-b:4,5-b’}dithiophen-2-yl)furan-2-yl)-5-(2-ethylhexyl)-3-(furan-2-yl)-4H thieno{3,4-c}pyrrole-4,6(5H)-dione, PTT = {2,2; 5′ 2″}-terthiophene-3-carbaldehyde, TTBO = 5,6-bis(octyloxy)-4,7-di(thieno{3}{3,2-b}thiophen-2-yl)benzo{c}{1,2,5}oxoadiazole, NH_2_-BDC = 2-amino terephthalic acid, PANI = polyaniline, p-PD = p-Phenylenediamine, IL = ionic liquid.

**Table 5:** Design and performances of selected IIP electrodes.

Template/Ligand/Monomers/Initiator	Synthesis Conditions	Final Ion Imprinted Material	Detection Technique	Performances (Water Source)	Year, Ref.
Vinylic polymers					
Mn(II)/1-(2-Pyridylazo)-2-naphthol/MAA & EGDMA/AIBN	Thermal radical polymerization at 60 °C, 24 h; acid wash for 24 h then coating on MWCNT-Chit-IL-modified GCE	Mn(II)-IIP/MWCNT-Chit-IL coated on GCE	SWASV in acetate buffer, pH 6. 1.0 mg IIP, 2 min preconcentration at −1.4 V	LOD: 0.15 µM; sensitivity 130.5 nA μM^−^^1^ cm^−^^2^). (Wastewater)	[133]
Pb(II)/2,2′:6′,6″-terpyridine/EGDMA/AIBN	Thermal polymerization at 60 °C, 24 h in DMF. 0.1 M HCl to remove Pb(II)	IIP-CPE-oil = 15/55/30%	DPASV in acetate buffer, pH 5. 6 min preconcentration at −1 V.	LOD: 0.11 nM; sensitivity 694 nA nM^−1^ cm^−2^) for Pb(II) in the 0.4–10 nM range. (Tap or well water)	[119]
Cu(II)/5-methyl-2-thiozylmethacrylamide/ EGDMA/AIBN	Thermal polymerization at 70 °C/12 h then 80 °C/3 h in DMSO. Cu(II) was removed in 0.5 M HNO_3_.	Carbon paste: Cu(II) IIP 20%/65% C/5% MWCNTs/Parrafin oil 10%	Potentiometric titration of Cu(II) in EDTA at pH 6	Cu selective electrode. LOD 4.0 × 10^−^^7^ M; stable at 4.0–8.0 pH range. Linear range: 1.0 × 10^−6^–1.0 × 10^−1^ M Cu(II); Sensitivity: 26.1 ± 0.9 mV/decade. Stable 1 year. (Tap or dam or river water)	[122]
Pb(II)/IA/EGDMA/AIBN	1 mmol Pb(ClO_4_)_2_ + 2 mmol IA in 35 mL CAN mixed for 30 min then 8 mmol EGDMA and 0.08 g AIBN added. Polymerization at 70 °C for 24 h. Pb(II) leached using EDTA.	CPE: IIP/MWCNT/graphite/oil = 7/6/74.8/12.2% *w*/*w*.	SWV in −0.7 to −0.2 V vs. calomel; and scan rate = 500 mVs^−1^, pH 5, preconcentration at −1 V for 60 s.	LOD = 3.8 pmol L^−1^; Linear range = 1.0 × 10^−11^–8.0 × 10^−8^ mol L^−1^. (Sea or river water).	[121]
Eu(III)/AM/EGDMA/AIBN	0.0125 mmol of EuCl_3_ in 30 μL methanol+ 0.05 mmol AM in 0.47 mL + sonication + 30 dwell time + addition of 0.5 mmol EGDMA and 0.04 mmol AIBN. 1.5 µL of solution dropped on MWCNT-coated SPE. UV-triggered photopolyerization for 3 h.	1.5 µL of template in monomer and AIBN solution was dropped on MWCNT-coated SPE. UV-triggered photopolyerization for 3 h. Eu(III) removed in 0.6 M HCl at −1 V vs. Ag/AgCl.	DPV: −1.2 V to −0.6 V vs. Ag/AgCl at pH 4.7; scan rate = 100 mV s^−1^; see reference for details. Response of the sensor using 3.0 × 10^−5^ mol L^−1^ Eu(III) is ~4 times higher for SPE/MWCNT-IIVP compared to SPE-IIVP.	LOD = 4.0 × 10^−8^ mol L^−1^; linear range = 1.0 × 10^−7^–1.0 × 10^−3^ mol L^−1^. 95% of original response 30 uses or 2 month storage in water. Change in response less than 5% in the presence of 30–200 fold excess metal ions. (River or lake water)	[124]
**Conjugated polymers**					
Hg(II), Pb^2+^ Cd^2+^ Cu^2+^/pyrrole-EDTA like	Oxidative electropolymerization in CH_3_CN + TBAP	Film/CD	SWV at pH 4.4 pre-concentration anodic time = 40 s at 0.4 V vs. SCE,; scan rate = 50 mV s^−1^.	Hg^2+^: LR = 510^−8^ to 5.10^−6^, LOD = 10^−7^; Pb^2+^: −10^−8^ to 10^−6^, LOD = 5.10^−10^ Cd^2+^: 10^−7^ to 10^−5^ LOD = 510^−7^; Cu^2+^: 510^−8^ to 2.510^−7^ LOD = 5.10^−9^ (Tap water)	[126]
Hg(II)/CMC/pyrrole	Electropolymerization aqueous solution in KCL	Film/GCE	SWASV at pH of 3, in the −1 to 1.25 V potential rang, pre-concentration time = 60 s E_red_ = −1 V/SCE Scan rate 50 mV·s^−1^	20–800 µg·L^−1^. LOD = 0.1 µg·L^−1^ (Ground or tap water)	[134]
Hg(II)/pyrrole	Aqueous medium + NaCl Chronoamperometry performed on diazonium-modified gold electrode decorated with ZnO nanorods	IIPPy@ZnO NRs film coated on Au	SWV method, in the −0.6 to 0.9 potential range; ZnO/Hg(II)-IIP electrodes incubated solutions of either mercury, cadmium, lead or copper ions for 20 min.	Sensitivity: 7.17 ± 0.15 μA/M; LOD: 10^−12^ M (Drinking water)	[135]
Pb(II)/L-Cys/AA/pyrrole	Electropolymerization by CA on SAW sensor gold electrode. Conditions: 0.9 V/SCE, in water, pyrrole:10^−2^ M, L-Cys or AA: 10^−4^ M, Pb^2+^: 10^−3^ M, LiClO4: 0.1 M.	Sensing imprinted L-Cys/PPy or AA/PPy	SWASV in a 0.1 M buffer solution with duration: 0.02 s, Amplitude: 2 mV, Pulse: 50 mV, −0.8 to 0 V vs. SCE potential range, Pb(II)/L-Cys/AA/pyrrole electrodes incubated solution for 20 min in solution of lead.	LOD in the picomolar regime. Pb(II) detected in Bousselem river = 14 μg/L. (River water)	[127]
Cu(II)/para-phenylene diamine	CV in H_2_SO_4_ 0.5 M, 10 mM of Cu^2+^ and 5 mM pPD on SPPtEs; 50 mV/s for 40 cycles.	Thin copper ion imprinted poly(para-phenylene diamine) films on SPPtED	DPV in the −0.2 V to + 0.6 V range, in acetate buffer pH 5.2,	LOD: 2.7 × 10^−9^; LR = 9.0 × 10^−10^–1.5 × 10^−8^, sensitivity = 1.30 μA nM^−1^ (Commercial drinking water)	[136]
**Sol-gel polymers**					
Cu(II)/TPDT	Complexation of Cu(II) by ligand-functionalized silane in ethanol followed by condensation of the silanols at reflux for 24 h in water/ethanol.	Carbon paste of diethylenetriamine-functionalized copper ion-imprinted silica gel.	DPSAV at pH 5.2, in the −0.8 to +0.8 V potential range, pre-concentration time = 1800 s at Ered = − 0.51 V vs. SCE; scan rate = 20 mV s^−1^.	LOD = 1.82 × 10^−7^ mmol L^−1^. No significant change in sensor response in the presence of Fe(II), Ni(II), Zn(II) or Pb(II). (Tap water)	[128]
Cd(II)/AAAPTS/ECH/TEOS	1 mmol of AAAPTS and 0.5 mmol CdCl_2_ mixed in 100 mL anhydrous ethanol, 1 h stirring and heating. Then 1 mmol of ECH added and stirring at 60 °C was conducted for 2 h. Finally 5 mmol TEOS and 2.5 mL NH_4_OH (14%) were added to the mixture under stirring and reactionleft to proceed for 12 h.Sol-gel material was washed with ethanol than in 30 mL HCl (1 mol/L) to remove Cd(II).	CPE: graphite powder (57–75% (*w*/*w*)), IISG (0–13% (*w*/*w*)) and paraffin oil (25% (*w*/*w*))	DPASV in the −1 to −0.4 V at pH 5, after 300 s accumulation in Cd(II) solution at −1.1 V vs. Ag/AgCl,	10% IISG in CPE, LOD = LOD is 0.15 μg·L^−1^, selective to Cd(II) in the presence of 30–100 fold excess competitive metal ions. (Dam or aqueduct or tap or river or wastewater).	[129]
Eu(III)/PTMOS/MTMOS/TEOS/HCl in ethanol	Mixture of 50 μL TEOS, 50 μL ethanol, 30 μL PTMOS, 28 μL of MTMOS, 10 μL of 1 × 10^−4^ mol L^−1^ HCl and 50 μL of water left for 2 h. deionized. 10 μL of 10 mmol L^−1^ Eu3+ added to 90 μL of this mixture to obtain PPC. 1.5 µL of PCC solution dropped on SPE-polycatechol and left to gelify. IISG washing with HCl to remove Eu(III) template.	SPE-polycatechol-IISG membrane.	DPV in buffer (pH 4.7) Eu(III): 3 × 10^−7^ to 10^−3^ M; accumulation at −0.2 V for 300 s; scan range: −1.2 to −0.6 V vs. Ag/AgCl; scan rate = 100 mV s^−^^1^; amplitude = 0.05 V.	LOD = 1.0 × 10^−7^ mol·L^−1^; linear range = 0.3–1000 μmol·L^−1^; selectivity over Ni^2+^, Co^2+^, Cu^2+^, Fe^3+^ or Gd^3+^ with 50–100 fold excess concentration. (Application to tap water, Greenlake water and Panlong river water).	[131]
Cd(II)/{MPS/TMSPMA/TEOS}/{VIN/TRIM/AIBN}	0.18 g of Cd(NO_3_)_2_4H_2_O in 10 mL of ethanol + 0.90 mL VIN + 1 mL MPS, 1.2 mL TMSPMA + 1.1 mL TRIM + 0.075 g AIBN. 10 min purge in N_2_, then addition of 2 mL TEOS dissolved in ethanol and 0.95 mL of NaOH pH(1 mol·L^−1^). Polymerization: 60 °C for 24 h in absence of oxygen. Template removed with HNO3 (1 mol·L^−1^).	CPE-ion-imprinted hybrid polymer (IIHP). 80 mg of graphite + 20 mg IIHP+ 1 mL of 0.1 M KCl. After 12 h drying, 85 μL mineral oil was added to obtain a compact paste.	Accumulation: 2000 μg/L^−1^ of Cd(II) at pH 1, −1.2 V vs. Ag/AgCl, for 300 s. DPASV in the −1 to −0.6 V in HCl 0.1 mol·L^−1^.	Linear ranges: Cd(II) in the 1 to 100 μg·L^−1^ and 2.75–5.0 mg·L^−1^. LOD = 0.10 µg·L^−1^. Recovery > 93.6% in rivers and drinking water (Peru and Brazil). No interference with other metal ions, except for Hg(II) at 50 fold excess. (Drinking or river water)	[132]
UO_2_(II)/QFS/TMOS	Pre-gel: 40 mmol TMOS + 12 mL of propanol + 0.4 mL of 0.02 M HCl refluxed at 70 °C for 3 h. Sol: TMOS/QFS mixture. 0.1 mL of 0.1 M TEA added to catalyse sol-gel synthesis for 48 h at RT and 24 h at 100 °C. Final imprinted powder was crushed.	CPE preparation: carbon powder (CP) + IISG + paraffin oil (55:15:30) (% *w*/*w*).	DPCSV in the −0.4–+0.4 V vs. Ag/AgCl; accumulation time = 5 min.	LOD = 3.07 × 10^−10^ mol·L^−1^; linear range = 2.0 × 10^−9^–3.0 × 10^−7^ mol·L^−1^. No competitive effect of other metal ions. (Application in tap, pond and waste waters).	[130]

AAAPTS: 3-(2-(2-aminoethylamino)ethylamino)propyl-tri methoxysilane; AA: acrylic acid; AM: acrylamide; CAN: acetonitrile; CD: carbon disk; CMC: Carboxy methyl cellulose; ECH: epichlorhydrin; FCN: K_3_Fe(CN)_6_, IA = itaconic acid; L-Cys: L-cystein; LR: linear range; MR: Methyl Red; MTMOS: methyltrimethoxysilane; NRs: nanorods; PPy-EDTA like: poly(N,N-ethylenebis(N-((3-(pyrrole-1-yl)propyl) carbamoyl) methyl)-glycine; pPD: p-phenylenediamine; PQC: platinum quartz crystal; PTMOS: Phenyltrimethoxysilane; SPPtEs: Platinum screen printed electrodes; TBAP: Tetra-n-butylammonium perchlorate; TMOS: Tetramethylorthosilicate; TMSPMA: 3-(trimethoxysilyl)propyl methacrylate; TPDT: N1-(3-(trimethoxysilyl)propyl)diethylenetriamine; VIN: 1-vinylimidazole.

**Table 6:** Synoptic table reporting shortlisted MIP-based electrochemical sensors for the detection of a range of bacteria. The MIPs were prepared using bacteria or their specific molecular or macromolecular compounds.

Target	Functional Monomer	Electrode Material	Polymerization Technique	Detection Method	Detection Medium/LOD	Ref.
*E. Coli*	TEOS	Gold	Sol-gel imprinting	EIS	Urine; 1 to 10^6^ cfu/mL LOD = 1 cfu/mL	[137]
*S. Aureus*	AP	Carbon	Electropolymerization	CV, EIS	Tap water LOD = 0.60 nM	[138]
*Aeromas hydrophila (AHLs)*	MAA, DMHF	Magnetic Glassy carbon	Controlled Radical polymerization	DPV	Solutions prepared in lab and spiked; 2.5.10^−9^ to 1.O.10^−7^ mol/L LOD = 8.10^−10^ mol/L	[141]
*Bacillus cereus* (spore)	Pyrrole	Carbon paste	Electropolymerization	CV	Solutions prepared in lab and spiked; 10^2^ to 10^5^ cfu/mL LOD = 10^2^ cfu/mL	[140]
*Proteus mirabilis* (flagella)	Phenol	Carbon	Electropolymerization	CV, EIS, SWV	Tap water LOD = 0.9 ng/mL	[139]
Sulfate-reducing bacteria	CS	ITO/graphene	Electrodeposition	EIS	Solution prepared in lab and spiked; 1 to 10^8^ cfu/mL LOD = 0.7.10^4^ cfu/mL	[63]

AP: 3-aminophenol; CS: Chitosan; DMHF: 2,5-dimethyl-4-hydroxy-3(2H)-furanone; MAA: methacrylic acid.

## Data Availability

Not applicable.
